# Supplementary material for: Reputations for treatment of outgroup members can prevent the emergence of political segregation in cooperative networks
Source: Nat Commun. 2023 Nov 24;14:7721. doi: 10.1038/s41467-023-43486-7 (PMC10674010; doi:10.1038/s41467-023-43486-7)
Supplement: Supplementary file 1 — Supplementary Information [file 41467_2023_43486_MOESM1_ESM.pdf]

Supporting Information for:

Reputations for treatment of outgroup members can prevent the emergence of political segregation in cooperative networks

Brent Simpson*, Bradley Montgomery, and David Melamed*

Direct Correspondence to Brent Simpson (bts@sc.edu) or David Melamed (melamed.9@osu.edu).

Supplementary Information Includes:

Figures S1 – S8
Tables S1 – S6
Supplementary Methods
Supplementary Note 1: Sensitivity Analyses
Supplementary Note 2

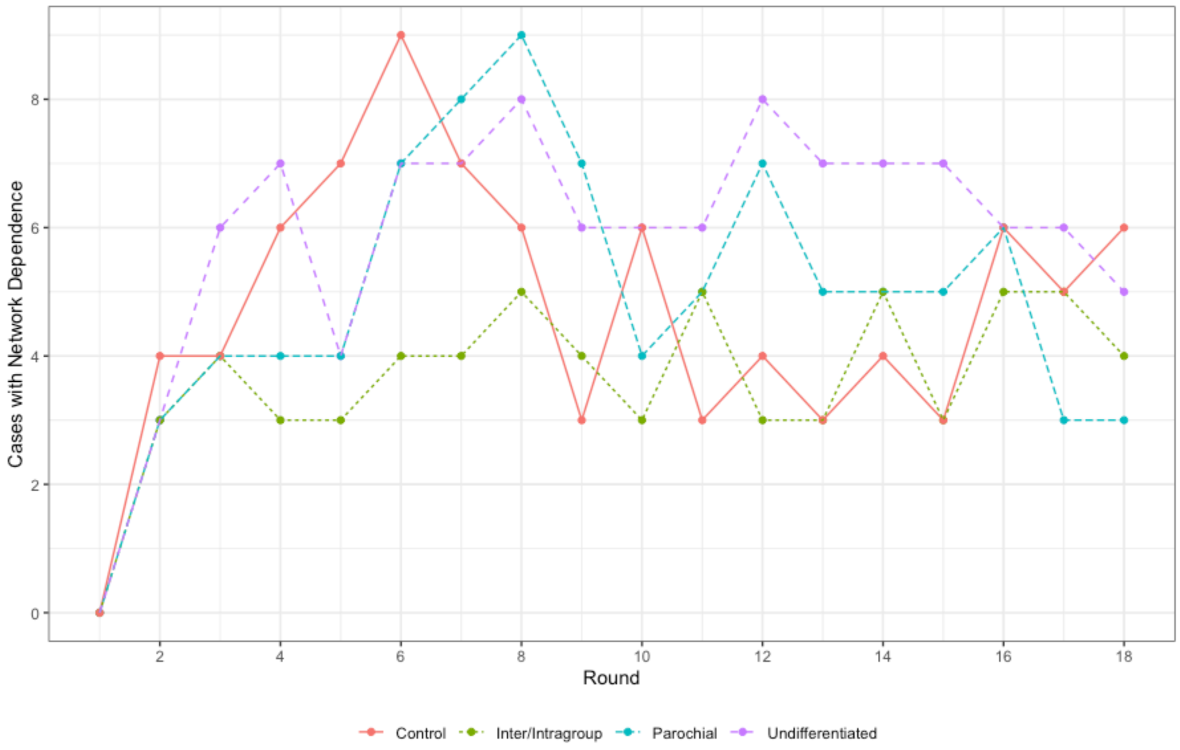

Figure S1: Count of network-rounds with significant network dependence on cooperation. Results derived from computing the association between neighbors' cooperation, i.e., the spatial correlation, and comparing it to a null distribution derived from permuting cooperation within network-rounds.

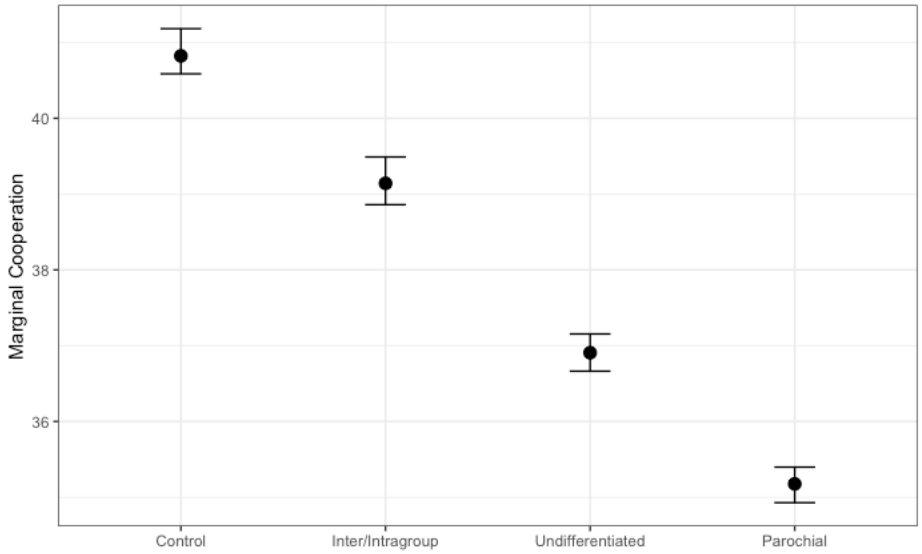

Figure S2: Marginal cooperation by experimental conditions for rounds 1-8. Margins drawn from Model 1 in Table 3 (N = 26,053 decisions nested in time nested in participants). Error bars are 95% confidence intervals computed via bootstrapping.

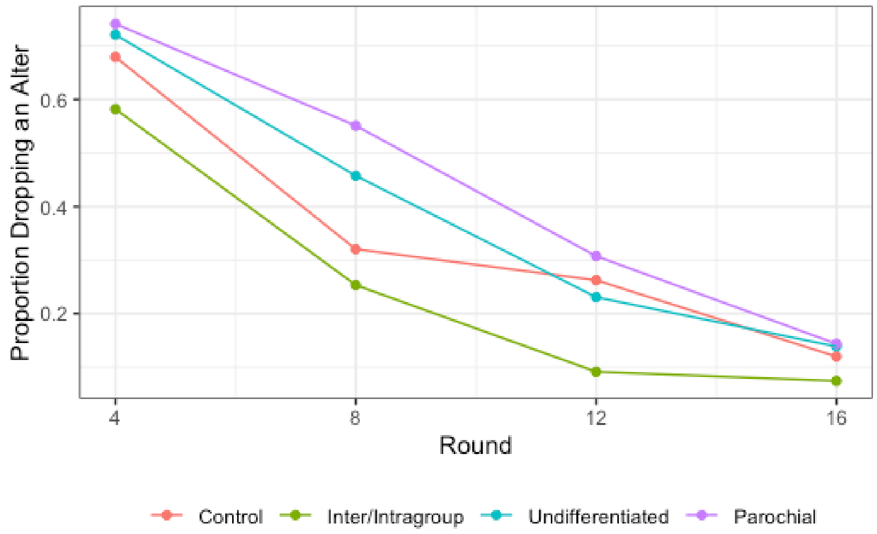

Figure S3: Proportion of participants who decided to drop an alter by round and experimental condition.

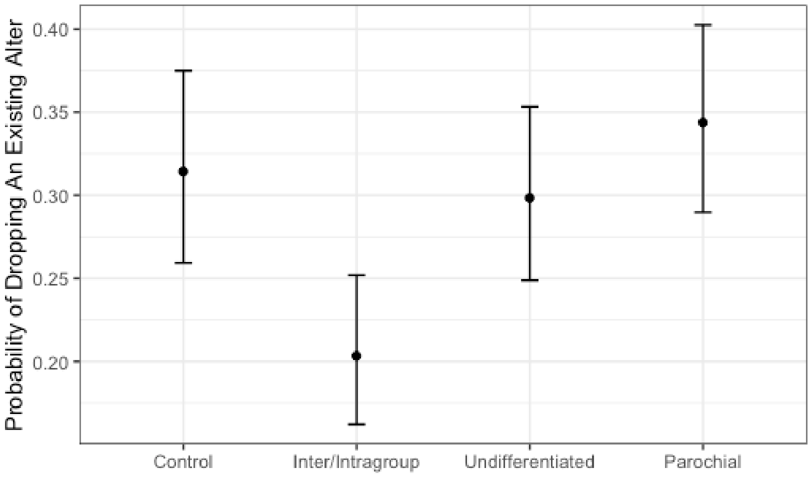

Figure S4: Marginal probability of dropping an alter by experimental condition for all four network dynamic phases. Margins drawn from Model 1, Table S1 (N is 3,651 binary decisions nested in 967 participants, nested in 40 networks). Error bars are 95% confidence intervals computed via bootstrapping.

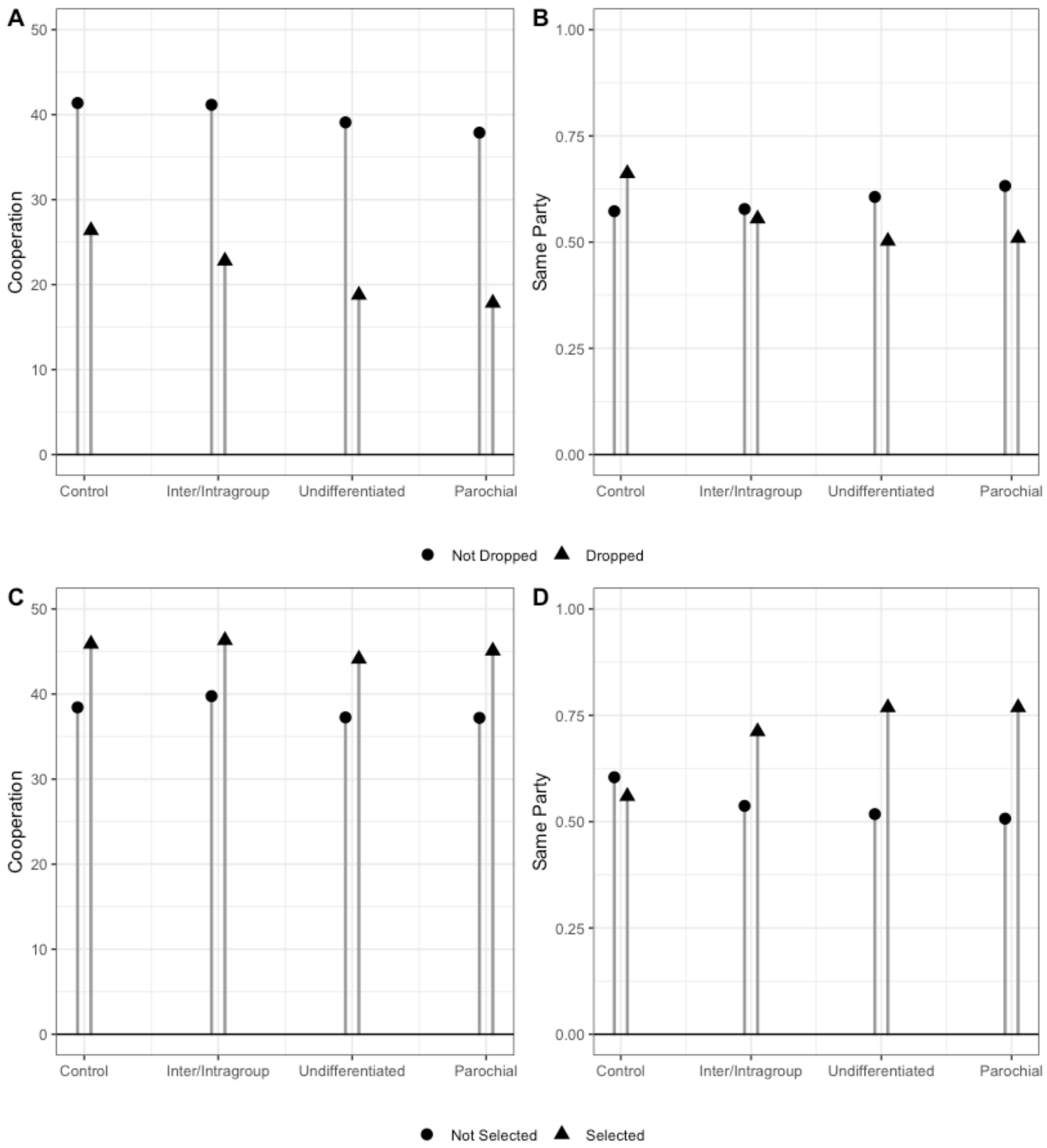

Figure S5: Average Cooperation/MUs given (A) and Same Party Ties (B) by whether the alter was selected to be dropped. Average Cooperation/MUs given (C) and Same Party Ties (D) by whether the alter selected to form a new tie.

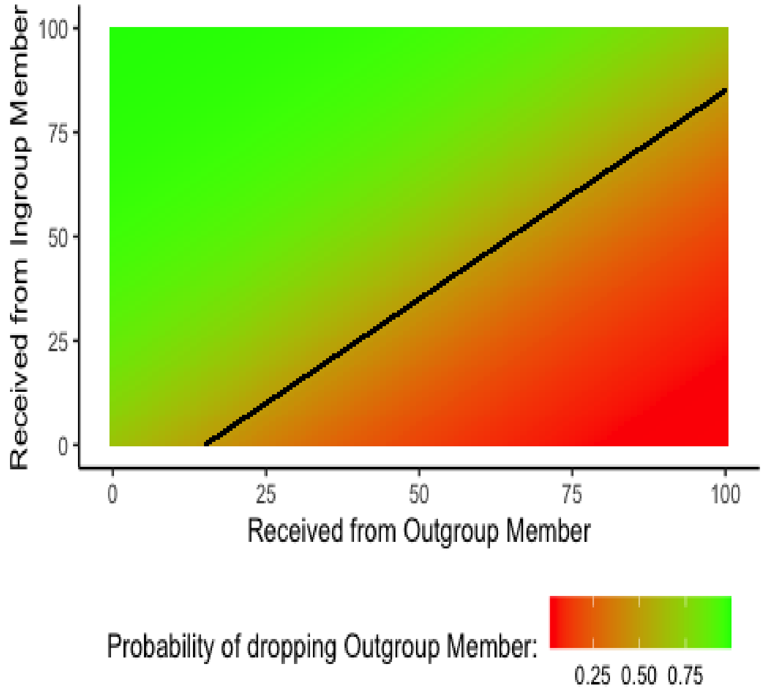

Figure S6: Probability of dropping the outgroup member when deciding between a single ingroup and outgroup member in the parochial reputation condition. Estimates drawn from Model 2 in Table S2. Reference line denotes probabilities of .5.

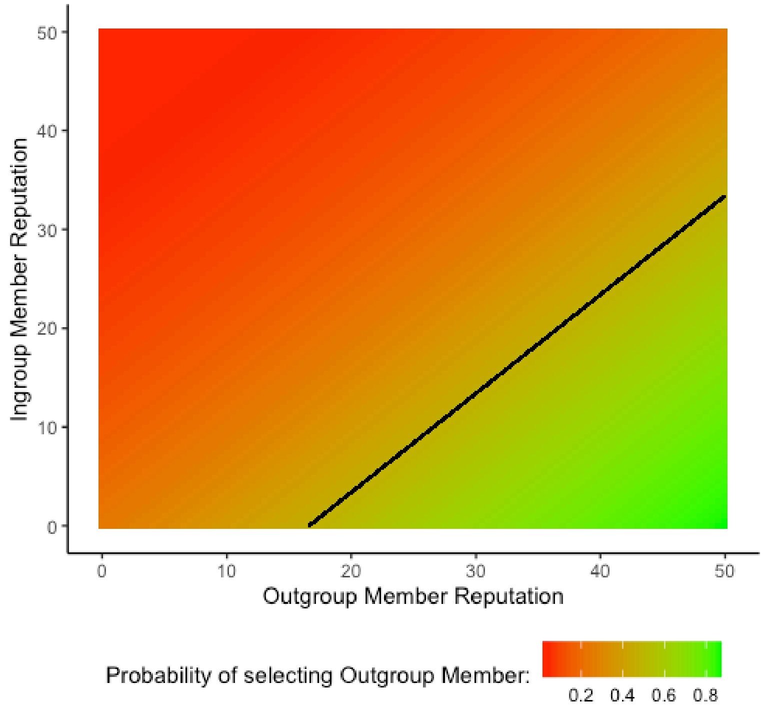

Figure S7: Probability of selecting the outgroup member when deciding between a single ingroup and outgroup member in the undifferentiated reputations condition. Estimates drawn from Model 2 in Table S3. Reference line denotes probabilities of .5.

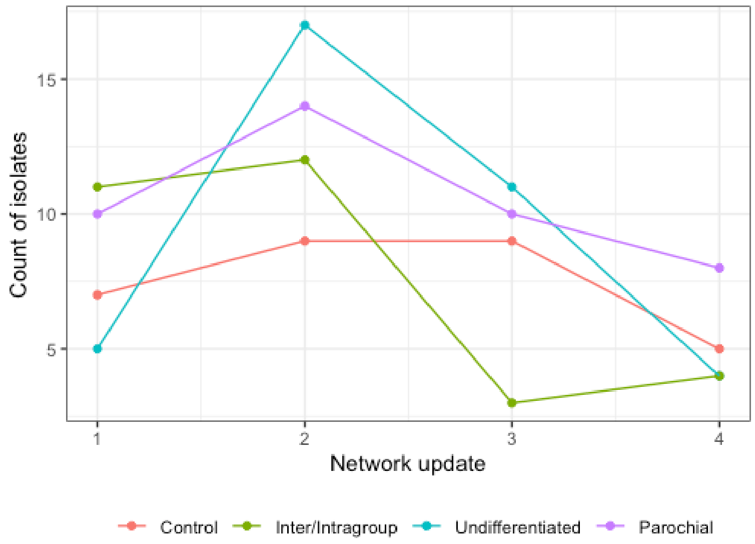

Figure S8: Count of participants who became isolated from the network for each network update by experimental condition.

Table S1: Mixed effects logistic regression models predicting whether the participant dropped an alter. Permutation-based *p*-value is reported in parentheses.

Parochial Reputations[1]	0.133
	(.999)
Inter/Intragroup Reputations[1]	-0.585
	(.031)
Undifferentiated[1]	-0.075
	(<.001)
Same Party	-0.203
	(.055)
Number of Alters	0.373
	(<.001)
Amount Received	-1.333
	(<.001)
Amount Given	-0.249
	(.001)
Following Round 8[2]	-1.136
	(.999)
Following Round 12[2]	-1.896
	(.999)
Following Round 16[2]	-2.630
	(.999)
Constant	-0.752
	(<.001)

Note: Inference is based on 1,000 permutations of the outcome within network rounds. [1]Reference category is the Control condition. [2]Following Round 4 is the reference category. N is 3,651 binary decisions nested in 967 participants, nested in 40 networks. All tests are two-tailed.

Table S2: Summary of conditional logistic regression models predicting which alter the participant dropped. Robust standard errors are reported in brackets and parametric *p*-values are reported in parentheses.

	Model 1		Model 2	
	Log Odds Coeff.	Odds Ratio	Log Odds Coeff.	Odds Ratio
	[Robust S.E.]	(*p*-value)	[Robust S.E.]	(*p*-value)
	Lower Limit 95% C.I.	Upper Limit 95% C.I.	Lower Limit 95% C.I.	Upper Limit 95% C.I.
Alter Endowment	.317	1.373	.327	1.387
	[.039]	(<.001)	[.040]	(<.001)
	.240 – .394		.249 – .406	
Received from Alter	-.048	.953	-.048	.953
	[.003]	(<.001)	[.003]	(<.001)
	-.054 – -.042		-.054 – -.042	
Same Party (S)	-.373	.689	.211	1.235
	[.093]	(<.001)	[.231]	(.263)
	-.555 – -.190		-.242 – .664	
Average Amount Given	-.051	.951	-.051	.950
	[.005]	(<.001)	[.005]	(<.001)
	-.060 – -.041		-.061 – -.041	
S × Parochial Reputations			-.914	.401
			[.283]	(.002)
			-1.469 – -.359	
S × No Politics/Control Condition			-.576	.562
			[.287]	(.053)
			-1.138 – -.014	
S × Intra/Intergroup Reputations			-.544	.580
			[.317]	(.091)
			-1.165 – .077	

Note: 1,251 alters dropped out of 5,928 alternatives. All tests are two-tailed.

Table S3: Summary of conditional logistic regression models predicting with whom the participant chose to form a tie. Permutation-based *p*-value is reported in parentheses.

	Control	Undifferentiated	Parochial	Inter/Intragroup Reputations
Alter Endowment	0.369<br>(<.001)	0.102<br>(.052)	0.235<br>(<.001)	0.275<br>(<.001)
Average Prospective Alter Gave to Everyone	0.084<br>(<.001)	0.068<br>(<.001)		
Average Prospective Alter Gave to Their Own Ingroup Members			0.063<br>(<.001)	0.037<br>(.001)
Average Prospective Alter Gave to their Outgroup Members				0.076<br>(<.001)
Same Party Other	-.050<br>(.331)	1.214<br>(<.001)	1.445<br>(<.001)	0.958<br>(<.001)

Note: Inference is based on 1,000 permutations of the outcome within network-rounds. N in the Control condition is 268 selections out of 4,612 alternatives. N in the Undifferentiated condition is 380 selections out of 7,745 alternatives. N in the Parochial condition is 412 selections out of 7,711 alternatives. N in the Inter/Intragroup condition is 208 selections out of 3,062 alternatives. All tests are two-tailed.

Table S4: Summary of mixed effects logistic regression model predicting whether the respondent accepted tie requests. Multiple requests nested in participants, and participants nested in networks. Permutation-based *p*-value is reported in parentheses.

Following Round 8[1]	-0.065
	(.491)
Following Round 12[1]	-0.365
	(.076)
Following Round 16[1]	-0.112
	(.444)
Average Given to Alters	0.084
	(<.001)
Same Party (S)	0.075
	(.459)
Parochial Reputations[2] (P)	-0.790
	(.056)
Inter/Intragroup Reputations[2] (I)	0.092
	(.269)
No Politics/Control[2] (C)	-0.058
	(.533)
S × P	0.520
	(.052)
S × I	-0.403
	(.138)
S × C	-0.329
	(.252)
Constant	-1.506
	(<.001)

Note: Inference is based on 1,000 permutations of the outcome within networks. [1]Following Round 4 is the reference category. [2]Undifferentiated is the reference category. N = 1,290 decisions nested in 40 networks. All tests are two-tailed.

Table S5: Summary of Cox Proportional Hazard Model predicting network isolation. Permutation-based *p*-value is reported in parentheses.

Endowment[1]	-0.001
	(<.001)
Average Given[1]	-0.087
	(<.001)
Parochial Reputations[2]	0.061
	(.993)
Inter/Intragroup Reputations[2]	0.240
	(<.001)
No Politics/Control[2]	0.206
	(<.001)

Note: Inference is based on 1,000 permutations of time to isolation within networks. [1]Time-varying covariate. [2]Undifferentiated reputations is the reference category. N is 139 isolates out of 3,617 rounds of exposure. All tests are two-tailed.

Table S6: Drop-outs by experimental condition and participant political affiliation.

	Control	Parochial	Inter/Intragroup	Undifferentiated
Overall	11	19	26	23
Democrats		11	12	11
Republicans		8	14	12

Supplementary Methods

This section contains screenshots of the experiment, including study instructions.

Study Instructions for the Control Condition

Welcome! In today's study, you will be making decisions with other participants.

Both your own decisions and the decisions of the others will influence your earnings. **Therefore, it is very important that you read all instructions carefully. After you have read the instructions, there will be a quiz. You need to pass the quiz to complete the study.**

At the beginning of the study, you will be connected to some of the other participants (Neighbors). Each participant will be given a letter identifier (e.g., U).

You and the neighbors you are connected to will make decisions in a series of rounds. These decisions will cause you and your neighbors to gain or lose points. You start with some initial amount of points.

You will also have an opportunity to change some of your neighbors throughout the study.

In addition to the $2.00 you recieve for participating, at the end of the study, you will also be paid a bonus of 1 cent for every 10 points in your account.

Next

Study Instructions for the Political Identity Visible Conditions

Welcome! In today's study, you will be making decisions with other participants.

Both your own decisions and the decisions of the others will influence your earnings. **Therefore, it is very important that you read all instructions carefully. After you have read the instructions, there will be a quiz. You need to pass the quiz to complete the study.**

At the beginning of the study, you will be connected to some of the other participants (Neighbors). Each participant will be given a letter identifier (e.g., U) and each participant will be color coded according to their political orientation (e.g. a red circle for Republicans and a blue circle for Democrats).

You and the neighbors you are connected to will make decisions in a series of rounds. These decisions will cause you and your neighbors to gain or lose points. You start with some initial amount of points.

You will also have an opportunity to change some of your neighbors throughout the study.

In addition to the $2.00 you recieve for participating, at the end of the study, you will also be paid a bonus of 1 cent for every 10 points in your account.

Next

Before the study begins, you will answer a few demographic questions. Specifically, we are interested in the participants' political orientations.

During the study, you will be able to identify other participants' political orientations based on the color of their circle. Those who identify more as a Democrat will be colored blue. Those who identify more as a Republican will be colored red.

Additionally, other participants will be able to identify your political orientations based on the color of your circle.

Continue

Giving Instructions for each condition

Specifically, in each round, **you will choose how many points (from 0 to 50, in 10-point increments) to give to each of the people you are connected to (i.e., your neighbors).** All participants will also choose how many points to give to each of the people they are connected to.

For *each* neighbor, the amount you Give will be doubled before it reaches your neighbor. For instance, if you choose to Give 20 points to one of your neighbors, that neighbor will receive 40 points. If you choose to Give no points, you do not pay any points and you do not change the points of that neighbor.

Each neighbor that you are connected to has the same choices with each of the neighbors they are connected to, including you. Any amount that the neighbor chooses to Give will be doubled before it reaches their neighbor. For instance, if they choose to Give 20 points to you, you will receive 40 points. If the neighbor chooses to Give no points, you won't gain any points.

Once everyone makes their decisions, the results are displayed. You will be shown the choices of each neighbor you are connected to, and how many points in total you gained or lost.

Remember, for every 10 points you have at the end of the game, we will add 1 cent to your bonus.

Next

Requesting Ties Instructions for the Control Condition and the Undifferentiated Reputations Condition

In those rounds that you are able to, if you choose to stop interacting with one of your neighbors, **you will choose which one new potential neighbor you want to connect with.**

Specifically, you will see a list containing the Participant IDs of all of the new neighbors with whom you could potentially connect. You will *also* see **how many points they have and the average number of points they gave to their neighbors in the last three rounds.** From the list, you can select one new potential neighbor to connect to.

The potential neighbor must confirm to be your neighbor as well in order to finalize it. Thus, the potential neighbor will see how many points you have at that point in the study and the average number of points you have given to your neighbors. The potential neighbor will be asked if they would like to connect with you. If they do, you will be successfully connected with the new neighbor.

Similarly, you will be given an opportunity to confirm any connection others want to form with you. You will see the number of points they have at that point in the study and the average number of points they gave to their neighbors.

Be sure you have carefully read the instructions. Next, you will complete a few questions to ensure your understanding of the task.

Next

Requesting Ties Instructions for the Parochial Reputations Condition

In those rounds that you are able to, if you choose to stop interacting with one of your neighbors, **you will choose which one new potential neighbor you want to connect with.**

Specifically, you will see a list containing the Participant IDs of all of the new neighbors with whom you could potentially connect. You will *also* see **how many points they have and the average number of points they gave to their neighbors with the same political orientation in the last three rounds.** From the list, you can select one new potential neighbor to connect to.

The potential neighbor must confirm to be your neighbor as well in order to finalize it. Thus, the potential neighbor will see how many points you have at that point in the study and the average number of points you have given to your neighbors with the same political orientation. The potential neighbor will be asked if they would like to connect with you. If they do, you will be successfully connected with the new neighbor.

Similarly, you will be given an opportunity to confirm any connection others want to form with you. You will see the number of points they have at that point in the study and the average number of points they gave to their neighbors with the same political orientation.

Be sure you have carefully read the instructions. Next, you will complete a few questions to ensure your understanding of the task.

Next

Requesting Ties Instructions for the Intra/Intergroup Reputations Condition

In those rounds that you are able to, if you choose to stop interacting with one of your neighbors, **you will choose which one new potential neighbor you want to connect with.**

Specifically, you will see a list containing the Participant IDs of all of the new neighbors with whom you could potentially connect. You will *also* see **how many points they have, the average number of points they gave to their neighbors with the same political orientation in the last three rounds, and the average number of points they gave to their neighbors with a different political orientation in the last three rounds.** From the list, you can select one new potential neighbor to connect to.

The potential neighbor must confirm to be your neighbor as well in order to finalize it. Thus, the potential neighbor will see how many points you have, the average number of points you gave to your neighbors with the same political orientation, and the average number of points you gave to your neighbors with a different political orientation. The potential neighbor will be asked if they would like to connect with you. If they do, you will be successfully connected with the new neighbor.

Similarly, you will be given an opportunity to confirm any connection others want to form with you. You will see how many points they have, the average number of points they gave to their neighbors with the same political orientation, and the average number of points they gave to their neighbors with a different political orientation.

Be sure you have carefully read the instructions. Next, you will complete a few questions to ensure your understanding of the task.

Next

Political Orientation Question for the Political Identity Visible Conditions

Before the study begins, you will answer a few demographic questions. Specifically, we are interested in the participants' political orientations.

During the study, you will be able to identify other participants' political orientations based on the color of their circle. Those who identify more as a Democrat will be colored blue. Those who identify more as a Republican will be colored red.

Additionally, other participants will be able to identify your political orientations based on the color of your circle.

1) Much closer to the Republican Party

2) Closer to the Republican Party

3) Somewhat closer to the Republican Party

4) Somewhat closer to the Democratic Party

5) Closer to the Democratic Party

6) Much Closer to the Democratic Party

Comprehension Check Questions

Be sure you have carefully read the instructions. Next, you will complete a few questions to ensure your understanding of the task.

If you click **Give 30** points to one neighbor

- **Your points do not change and the neighbor gains 60 points.**
- **You pay 30 points for the neighbor to gain 60 points.**
- **Your points and the neighbor's points do not change.**
- **You pay 30 points and the neighbor gets points based on how many points you have.**

Be sure you have carefully read the instructions. Next, you will complete a few questions to ensure your understanding of the task.

If you click **Give 0**

- **Your points do not change and the neighbor gains 100 points.**
- **You pay 50 points for the neighbor to gain 100 points.**
- **You pay 100 points for the neighbor to gain 100 points.**
- **Your points and the neighbor's points do not change.**

Be sure you have carefully read the instructions. Next, you will complete a few questions to ensure your understanding of the task.

Imagine that both you and a neighbor choose **Give 0**. What are your and the neighbor's final point earnings for the round?

- **You and the neighbor both gain 0 points.**
- **You gain 100 points and the neighbor loses 50 points.**
- **You lose 50 points and the neighbor gains 100 points.**
- **You and the neighbor both gain 100 points.**

Be sure you have carefully read the instructions. Next, you will complete a few questions to ensure your understanding of the task.

After you and your neighbors have made decisions and viewed the results, you will:

Always begin a new round of decisions with the same neighbors.

Periodically decide whether or not you want to stop interacting with one of your neighbors; otherwise, begin a new round of decisions with the same neighbors.

Always decide whether or not you want to stop interacting with one of your neighbors.

Be sure you have carefully read the instructions. Next, you will complete a few questions to ensure your understanding of the task.

What does the reputation score represent? In other words, how is the reputation score calculated?

average ratings by neighbors with the same political orientation over the last three rounds and average ratings by neighbors with a different political orientation over the last three rounds.

average number of points given to neighbors with the same political orientation and average number of points given to neighbors with a different political orientation.

average ratings by neighbors with the same political orientation over the last three rounds.

average number of points given to neighbors with the same political orientation.

average ratings by neighbors over the last three rounds.

average number of points given.

Giving Round in the Control Condition

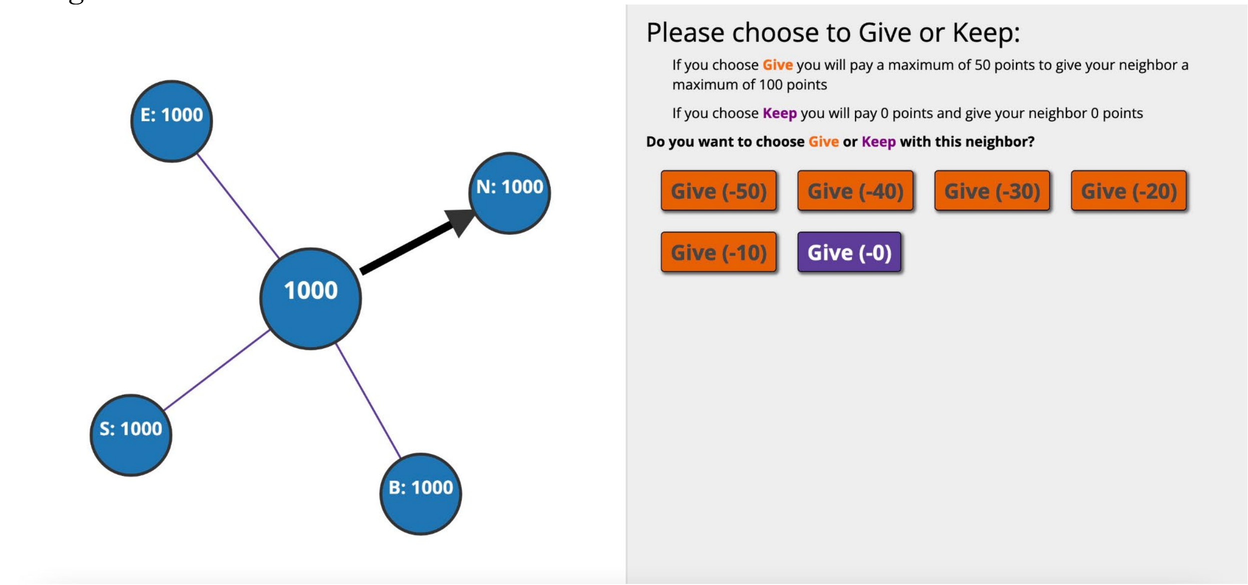

Giving Round in the Political Identity Visible Conditions

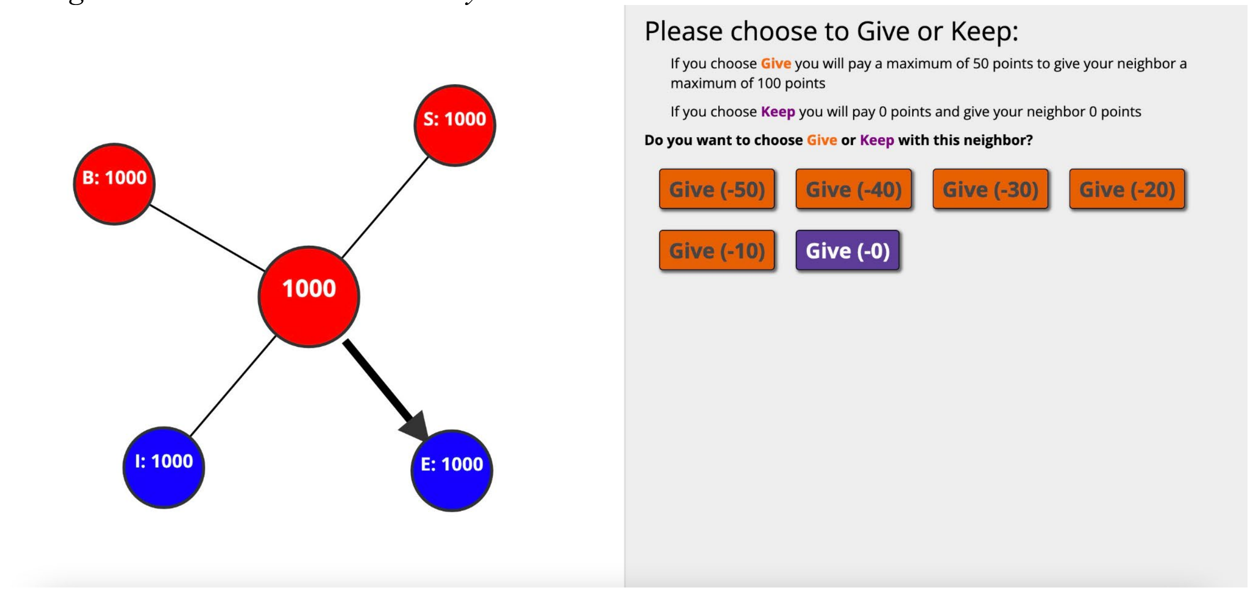

Results Screen after Giving Round

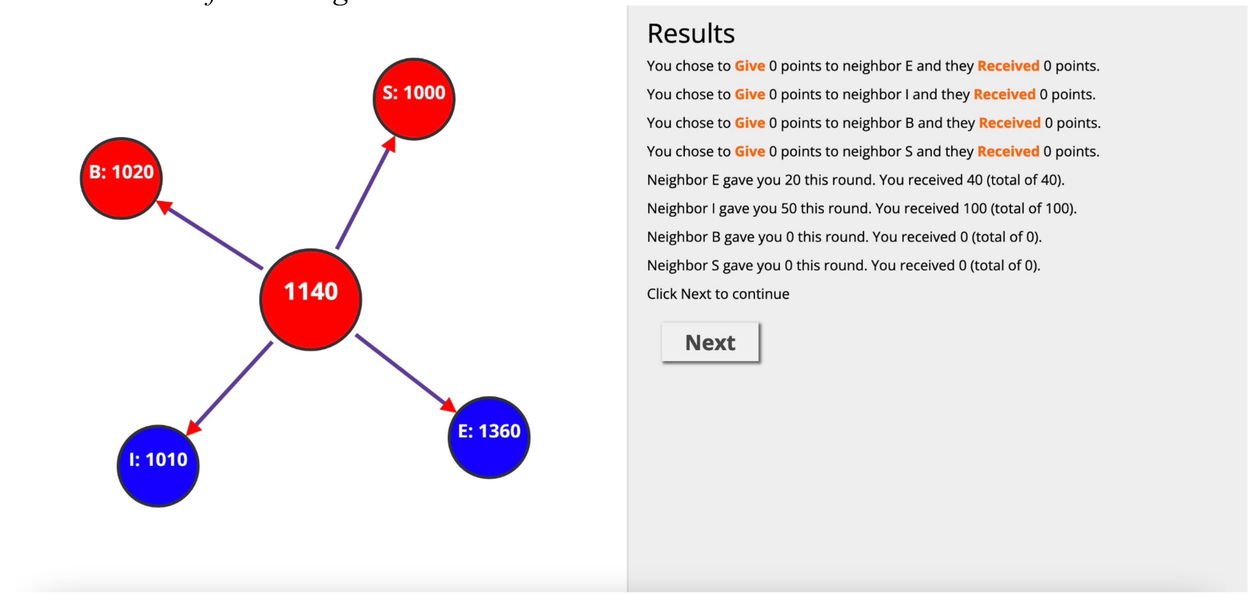

Tie Cutting Phase for the Control Condition (the color of the potential alters does not represent political orientation in this condition)

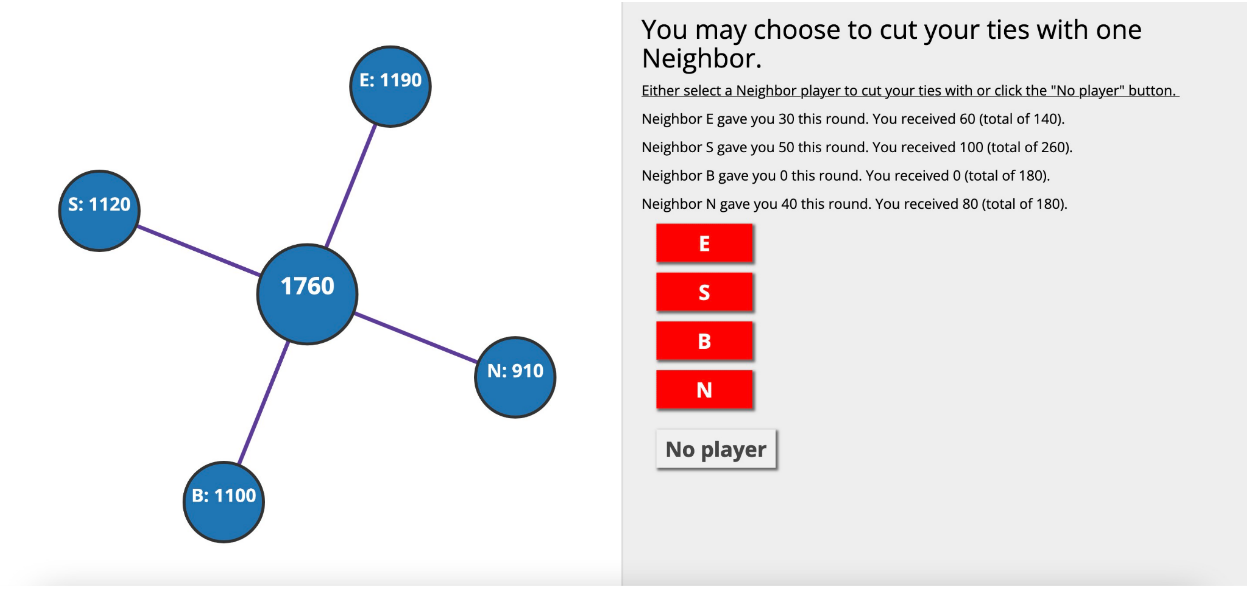

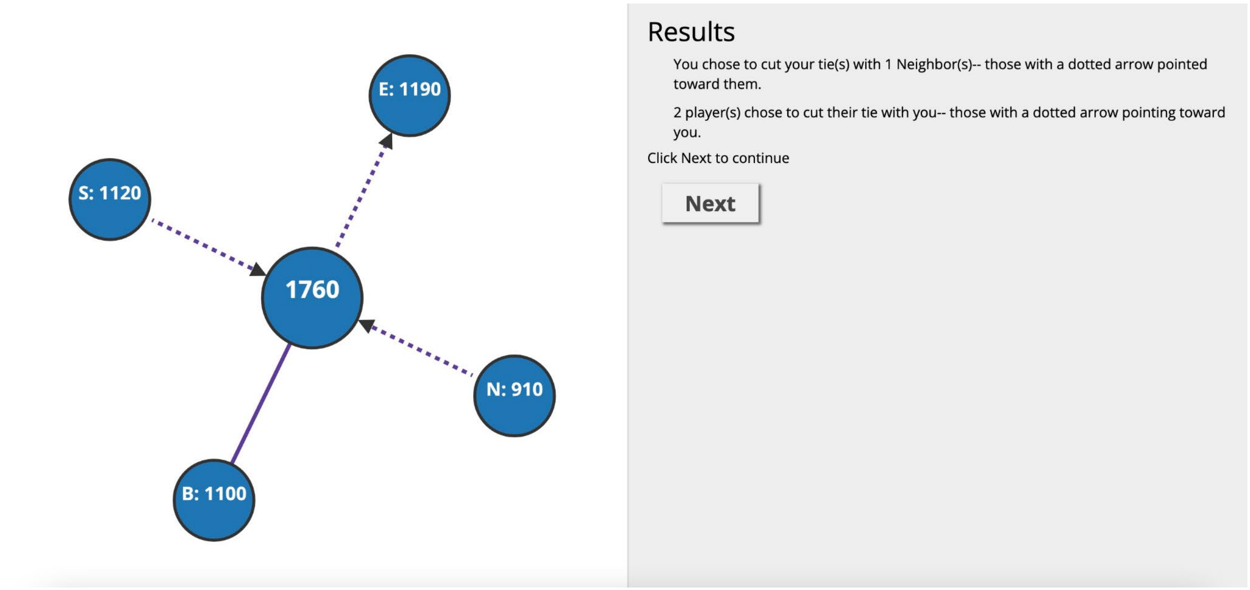

Tie Cutting Phase for the Political Identity Visible Conditions

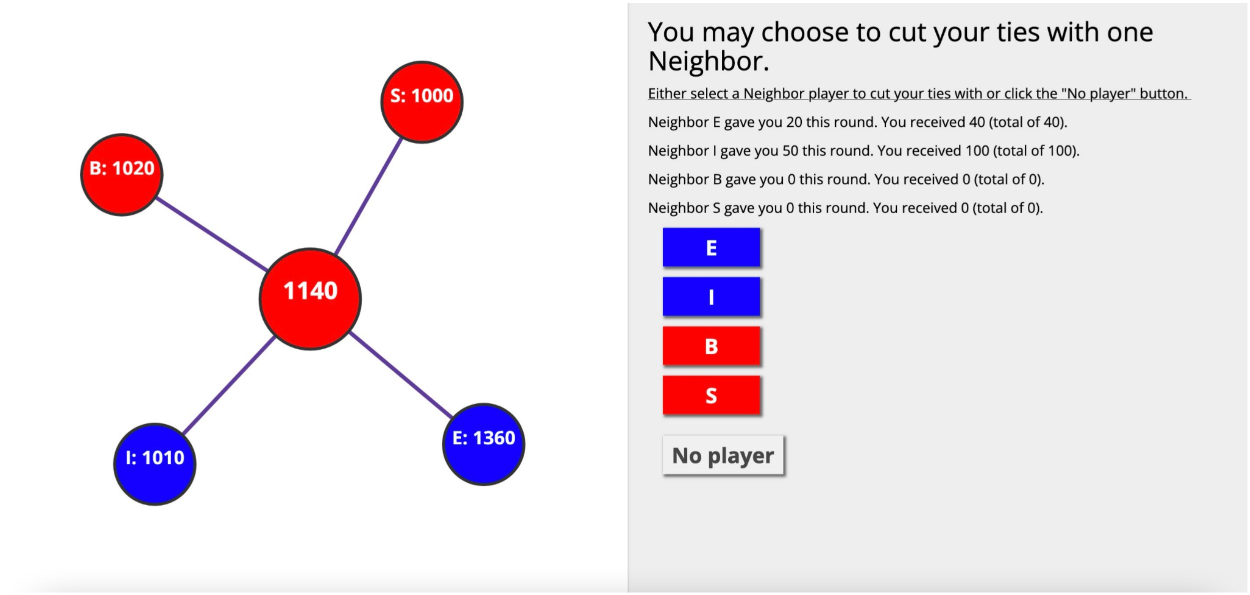

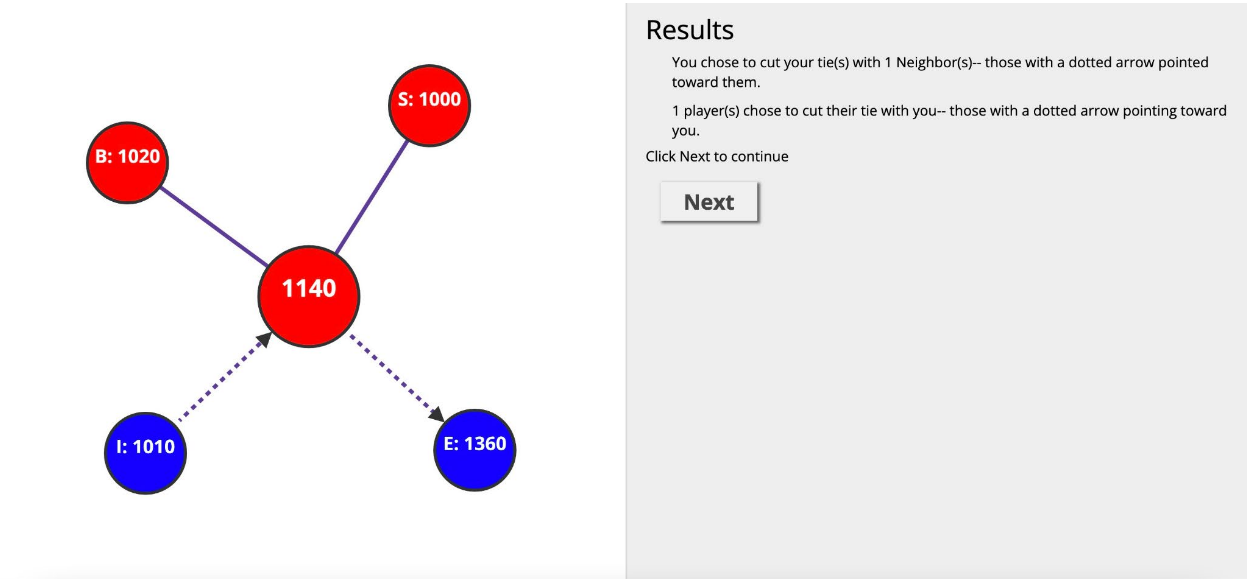

Requesting Tie Phase for the Control Condition (the red coloring of the potential alters does not represent political orientation in this condition)

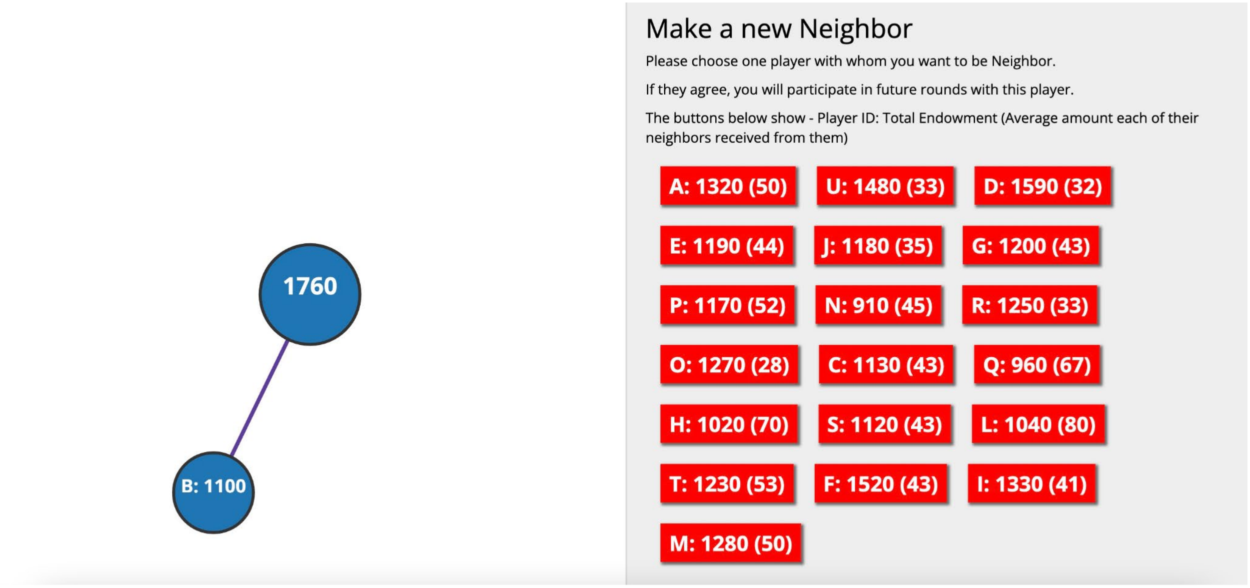

Requesting Tie Phase for the Undifferentiated Reputations Condition

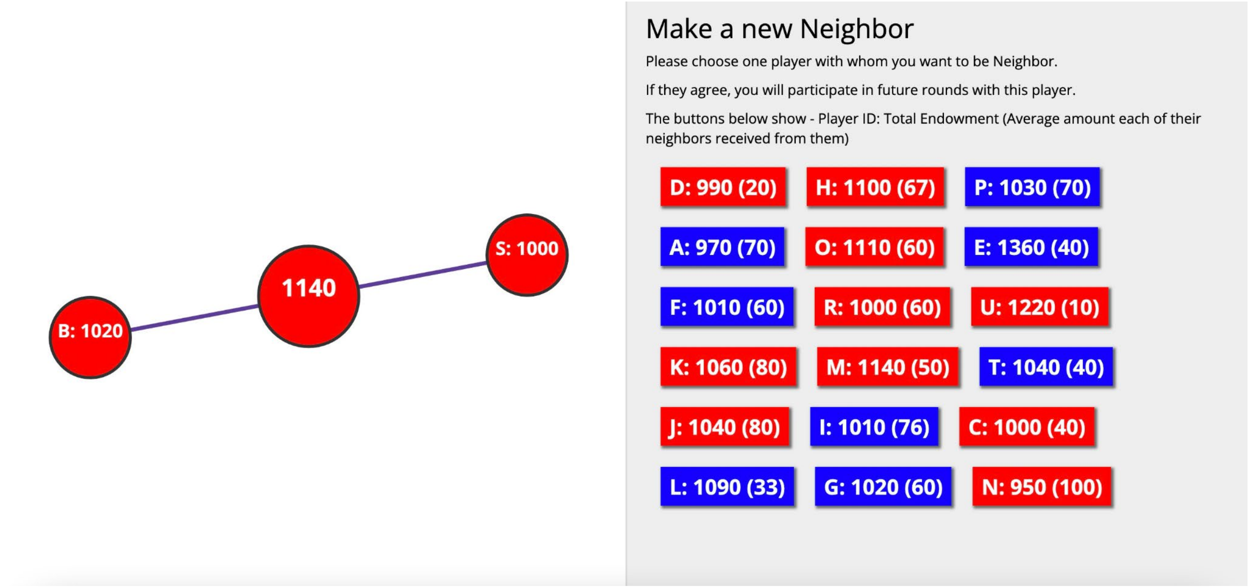

Requesting Tie Phase for the Parochial Reputations Condition

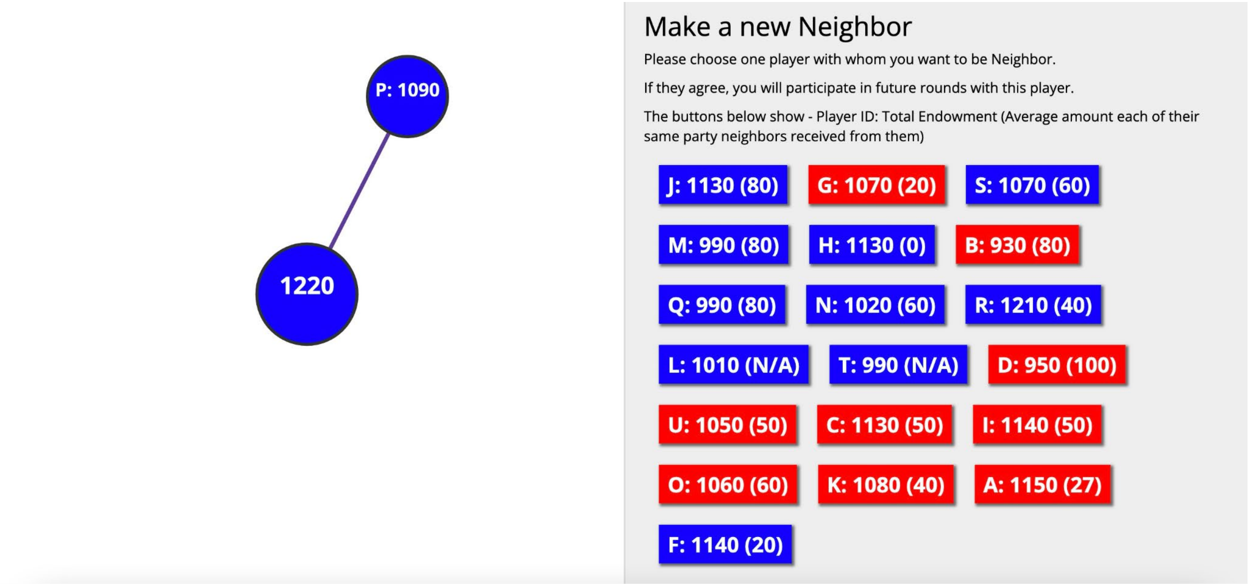

Requesting Tie Phase for the Intra/Intergroup Reputations Condition

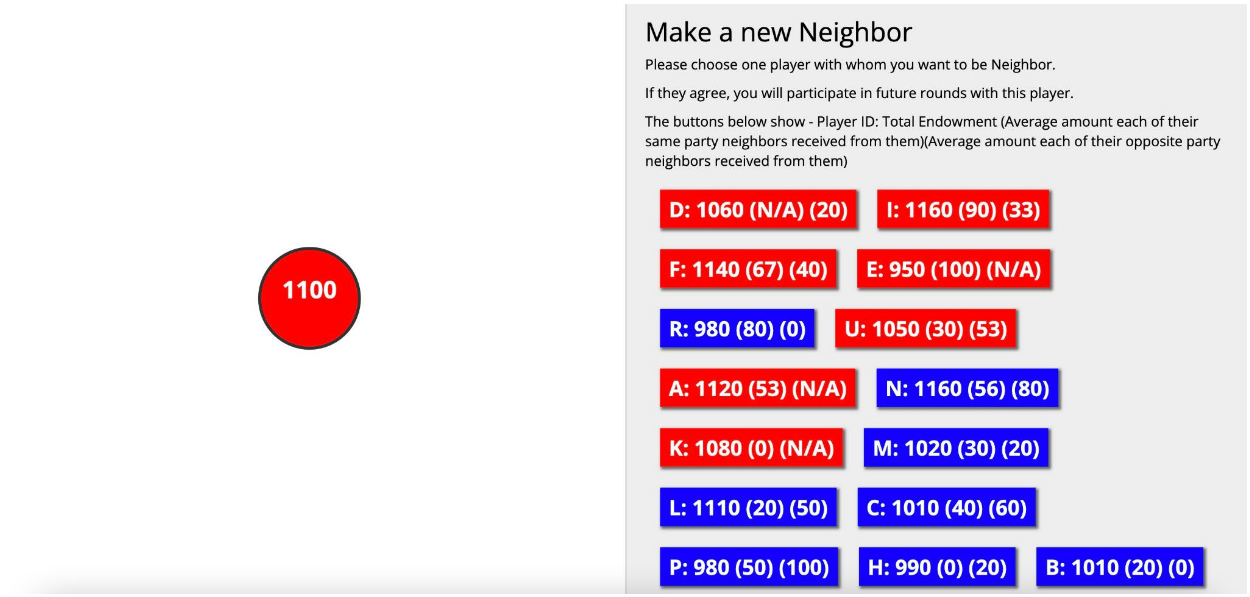

Tie Approval/Rejection Phase

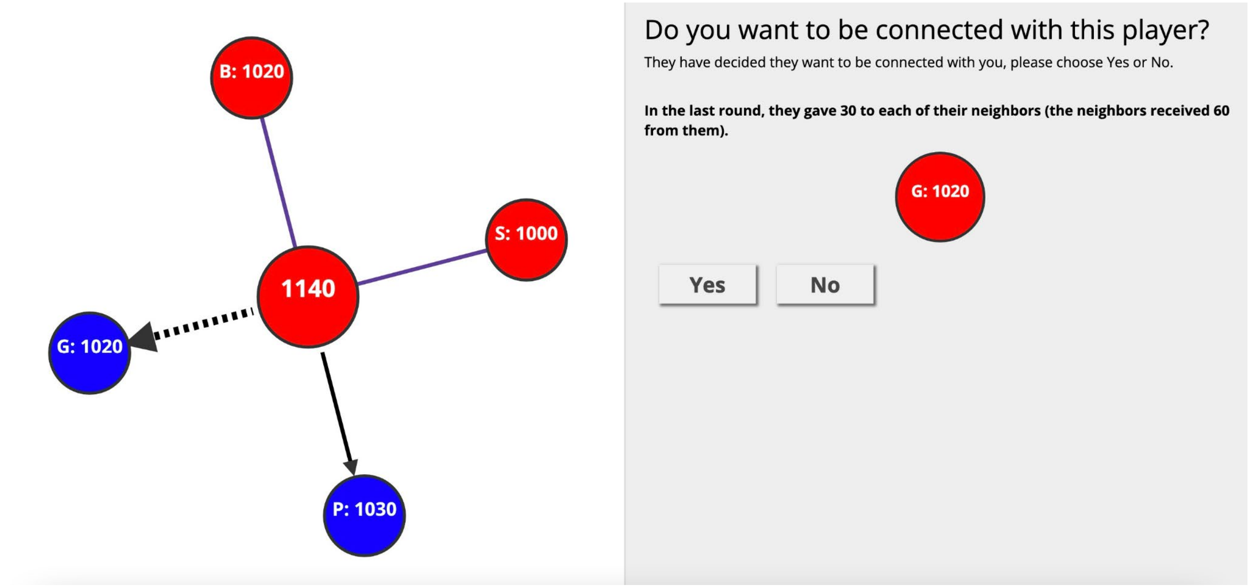

Supplementary Note 1
Sensitivity Analyses
In this section we report sensitivity analyses illustrating how the results described above and in the main text vary by the self-identified political identities of participants, gender, and whether the participants rated themselves as “much closer” to the Republican or Democratic party (i.e., a 1or 6) on our six-point scale. We refer to those participants who rated themselves at the extremes of our political identification scale as Strong Party Identifiers. The sample was 33% self-identified Republicans. Strong Party Identifiers accounted for 35% of the sample, with 22% of Republicans and 41% of Democrats reporting values at the ends on our political identification scale. The sample was also 50.2% women. While there are some subtle differences below, the bulk of the results reported below are consistent with the patterns noted above and in the main text.

Cooperation
Figure S9 shows average cooperation by round, participant political affiliation, and whether the participant is interacting with similar or dissimilar politically affiliated partners. A same affiliation effect on cooperation is apparent in rounds 1-4, with the lighter lines being higher than the darker lines. In rounds 5-8, Democrats and Republicans give outgroup members about the same (dark lines). But Republicans give other Republicans less than Democrats give other Democrats (grey lines).

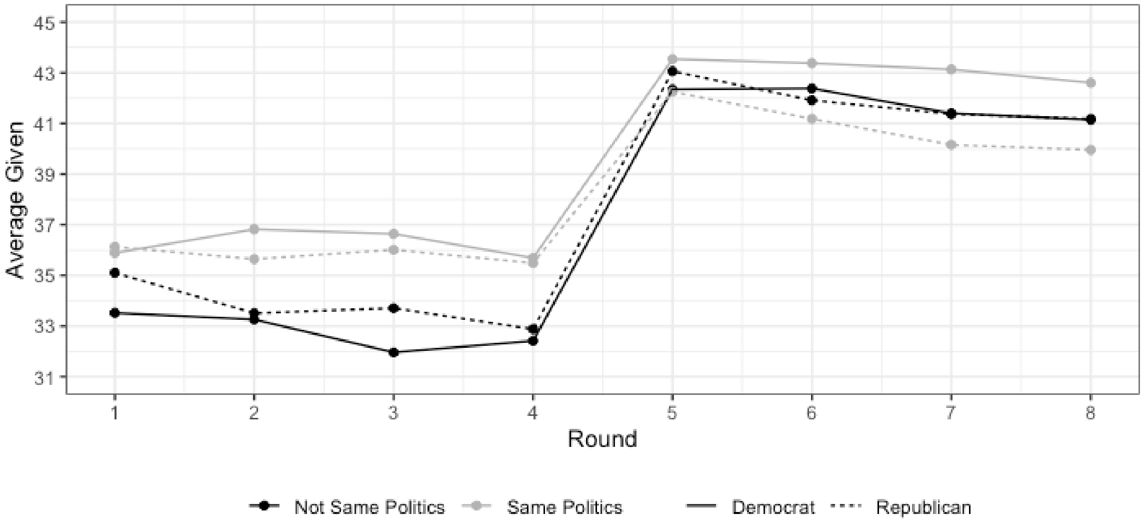

Figure S9: Average amount given by round. Line color denotes giving to others who are the same (light grey) or different (black) on politics. Solid lines denote Democrat respondents and dashed lines denote Republican respondents.

Similarly, Figure S10 shows average cooperation by political score, and by whether alter shared or did not share political affiliations. We observe higher rates of giving to same affiliated others for every politics score except for 3. While classified as “Republicans” in our study, these participants gave slightly more to Democrat partners than to Republican partners. It is also worth noting that Republican participants gave less on average. This affects network dynamics.

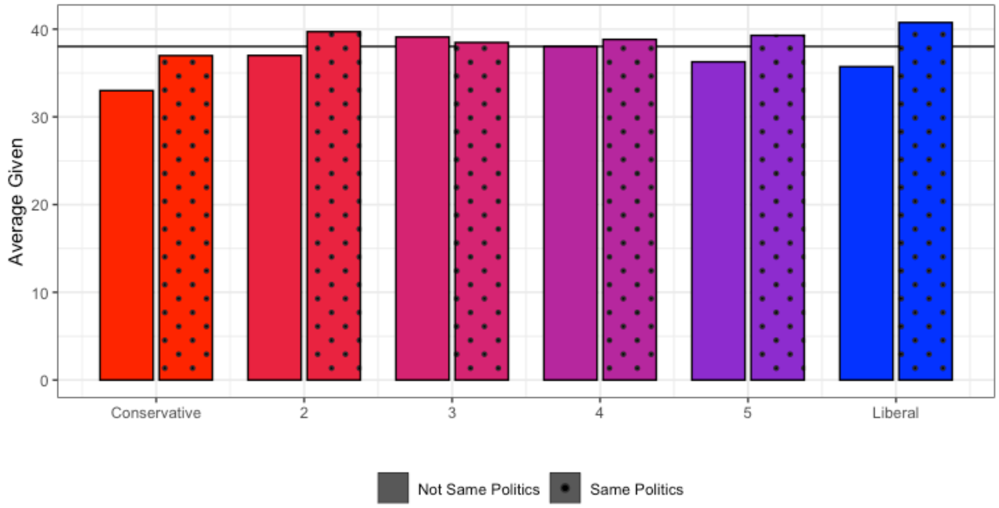

Figure S10: Average given in Rounds 1-8. X-axis and bar color denotes the Respondents score on the politics item. Solid bars denote interactions with alters who do not share a political identity, and dotted bars denote interactions with alters who share a political identity. The horizontal line is the average amount given overall in Rounds 1-8.

Figure S10 shows that ingroup favoritism in cooperation is stronger among Strong Party Identifiers, those who rated themselves as 1 or 6 on the six-point scale. Figure S11 illustrates this by experimental condition. In all three experimental conditions where politics were visible, Strong Party Identifiers gave the least to outgroup members. In the undifferentiated and inter/intragroup reputation conditions, outgroup animus is the only real result in S11. In the parochial condition, where only ingroup reputations are known, the effect of political similarity is enhanced: not only do Strong Party Identifiers give outgroup members less, they also give ingroup members more.

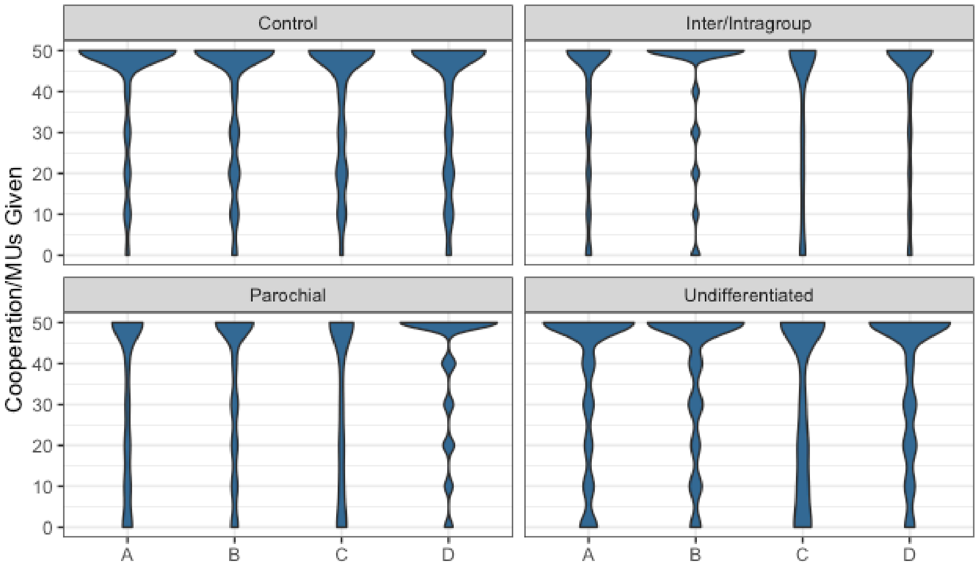

Figure S11: Violin plots of Given points for (A) Not Strong Identifiers interacting with others who were Not the Same Politics, (B) Not Strong Identifiers interacting with others who were the Same Politics, (C) Strong Identifiers interacting with others who were Not the Same Politics, and (D) Strong Identifiers interacting with others who were the Same Politics. Each condition shown.

Table S7 presents three linear mixed models predicting cooperation in rounds 1-8. Model 1 shows that there is an interaction between political orientation and same party and Figure S12 shows the implications of the interaction term: Republicans appear to give more to Democrats than Democrats give to Republicans. Model 2 shows that this effect is not significantly different between experimental conditions, and Model 3 shows that this effect (the interaction between politics and same party) becomes statistically insignificant once we control for direct reciprocity. It is not the case that Republicans give Democrats more than Democrats give Republicans; after we adjust for the lower overall levels of giving among Republicans, and therefore the lower levels of reciprocity towards them, the effect of same party on giving does not vary by participant politics.

Table S7: Summary of linear mixed models predicting how much the participant cooperated in rounds 1-8. Permutation-based *p*-value is reported in parentheses.

	Model 1	Model 2	Model 3
Parochial Reputations[1] (P)	-5.627	-7.832	-4.071
	(.999)	(.040)	(.999)
Intra/Intergroup Reputations[1] (I)	-1.681	-1.948	-.794
	(<.001)	(.158)	(.999)
Undifferentiated Reputations[1] (U)	-3.903	-4.653	-2.724
	(.999)	(.461)	(.999)
Same Party (S)	2.826	-.708	1.433
	(<.001)	(.162)	(<.001)
Republican Participant (R)	.518	.030	-.045
	(.028)	(.481)	(.478)
R × S	-.811	.388	-.315
	(.013)	(.507)	(.147)
R × P		.947	
		(.115)	
R × I		-.416	
		(.296)	
R × U		-.179	
		(.417)	
P × S		2.285	
		(.057)	
I × S		1.410	
		(.092)	
U × S		.767	
		(.464)	
R × P × S		-1.222	
		(.324)	
R × I × S		1.138	
		(.152)	
R × U × S		.008	
		(.436)	
Direct Reciprocity			.485
			(<.001)
Intercept	39.304	42.212	20.975
	(.999)	(.221)	(.999)
Variance Components			
Participant	11.034	.004	5.534
Network	3.722	4.184	2.214
AR(1)	.224	.059	.171

Note: Inference is based on 1,000 permutations of the outcome within network rounds.
[1]Reference category is the No Politics/Control condition. N = 31,442 decisions. All tests are two-tailed.

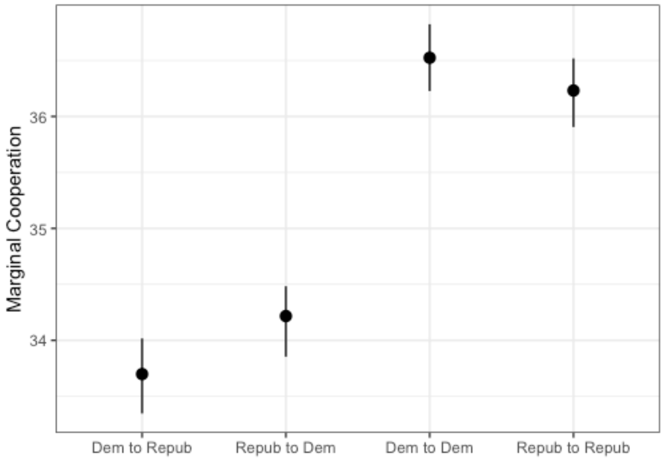

Figure S12: Marginal cooperation from Model 1 in Table S7 (N = 31,442 decisions). Error bars are 95% confidence intervals computed via bootstrapping.

Likewise, Table S8 presents two mixed models of cooperation that include indicators for whether participants were Strong Party Identifiers. Model 1 shows that Strong Party Identifiers do not give their same-party alters more than do participants who are non-Strong Party Identifiers (those who rated more moderately on the political identification scale), and Model 2 further shows that this does not vary by experimental condition. That is, while there is some descriptive evidence that our identity effects vary by political orientation and Strong Party Identification, neither of these patterns are detectable by our mixed models.

Table S8: Summary of linear mixed models predicting how much the participant cooperated in rounds 1-8. Permutation-based *p*-value is reported in parentheses.

	Model 1	Model 2
Parochial Reputations[1] (P)	-6.286	-7.540
	(.480)	(.099)
Inter/Intragroup Reputations[1] (I)	-1.113	-2.538
	(.503)	(<.001)
Undifferentiated Reputations[1] (U)	-4.217	-5.028
	(.520)	(.208)
Same Party (S)	.522	-1.051
	(.358)	(.046)
Strong Party Identifier (SPI)	-.107	-.726
	(.544)	(.150)
SPI × S	.021	.966
	(.588)	(.110)
SPI × P		.342
		(.337)
SPI × I		1.057
		(.041)
SPI × U		.719
		(.254)
P × S		2.165
		(.056)
I × S		2.109
		(.003)
U × S		1.391
		(.152)
SPI × P × S		-.833
		(.178)
SPI × I × S		-.956
		(.091)
SPI × U × S		-1.430
		(.109)
Intercept	41.540	42.515
	(.621)	(.068)
Variance Components		
Participant	.005	.004
Network	4.179	4.176
AR(1)	.059	.059

Note: Inference is based on 1,000 permutations of the outcome within network rounds.
[1]Reference category is the No Politics/Control condition. N = 31,442 decisions. All tests are two-tailed.

Turning to participant gender, Figure S13 shows average cooperation (MUs given) by whether alters were the same political affiliation. Both men and women give others from the same political party more. Men also gave more, on average. Figure S14 breaks this down by experimental condition. In the control condition, there is no statistically significant effect of political party, since politics was unknown in the control condition. But we observe men and women giving more to same party others in all other experimental conditions.

Table S9 presents two Models of cooperation in Rounds 1-8. The first model shows that men give more than women and that participants give more to same party others, and that the interaction between gender and same party others is not significant. Model 2 includes the three-way interaction between experimental condition, participant gender, and same party others. Two of the three terms corresponding to the three-way interaction are significant. Specifically, the effect of same party for men is weaker in the intra/intergroup and undifferentiated conditions than it is in the control condition. To illustrate, Figure S15 shows marginal or estimated cooperation from the regression model by each factor in the three-way interaction. While there are statistically significant differences in the effect of same party by experimental conditions, across experimental conditions we uniformly observe positive same party effects.

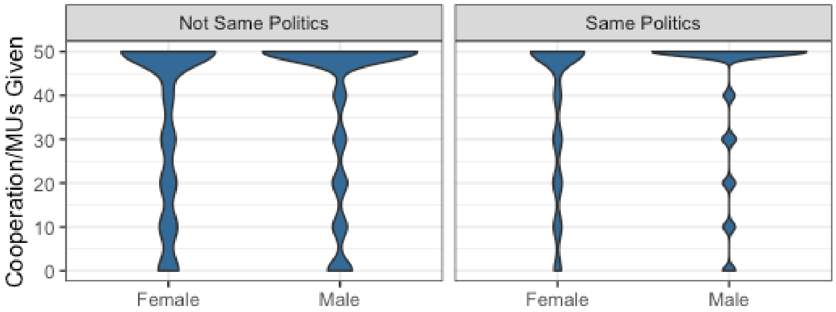

Figure S13: Violin plots of Cooperation (MUs given) by whether participant was interacting with same politics others and participant gender.

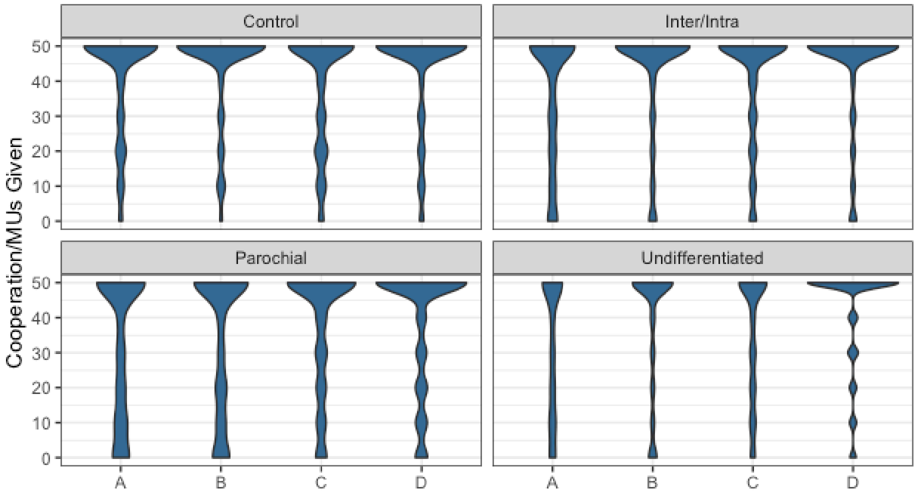

Figure S14: Violin plots of Cooperation (MUs given) for (A) Females interacting with others who were Not the Same Politics, (B) Males interacting with others who were Not the Same Politics, (C) Females interacting with others who were the Same Politics, and (D) Males interacting with others who were the Same Politics. Each condition shown.

Table S9: Summary of linear mixed models predicting how much the participant cooperated in rounds 1-8. Permutation-based *p*-value is reported in parentheses.

	Model 1	Model 2
Parochial Reputations[1] (P)	-6.369	-9.335
	(.018)	(<.001)
Inter/Intragroup Reputations[1] (I)	-1.971	-4.423
	(<.001)	(<.001)
Undifferentiated Reputations[1]	-4.556	-7.964
(U)	(<.001)	(<.001)
Same Party (S)	2.590	-1.389
	(<.001)	(.026)
Male Participant (M)	3.028	2.349
	(<.001)	(<.001)
M × S	-.078	1.434
	(.293)	(.071)
M × P		-1.200
		(.076)
M × I		1.916
		(.033)
M × U		2.042
		(.014)
P × S		6.393
		(<.001)
I × S		3.917
		(<.001)
U × S		4.621
		(<.001)
M × P × S		-.990
		(<.001)
M × I × S		-3.297
		(.007)
M × U × S		-1.986
		(.042)
Intercept	38.211	40.603
	(.999)	(.999)
Variance Components		
Participant	11.016	10.992
Network	3.635	3.652
AR(1)	.227	.228

Note: Inference is based on 1,000 permutations of the outcome within network rounds.
[1]Reference category is the No Politics/Control condition. N = 29,999 decisions. All tests are two-tailed.

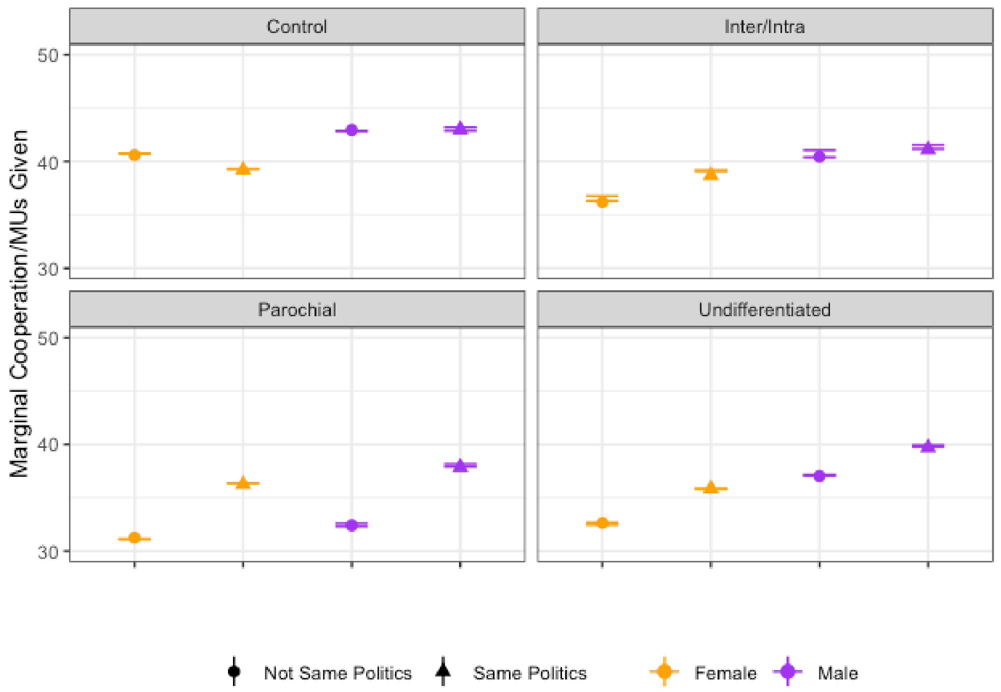

Figure S15: Marginal cooperation from Model 2 in Table S9 (N = 29,999 decisions). Error bars are 95% confidence intervals computed via bootstrapping.

Whether to drop an alter

Figure S16 shows how the proportion dropping alters varies by experimental condition and by self-reported political identification. Political identification appears unrelated to deciding to drop an alter. Figure S17 shows how the proportion dropping alters varies by experimental condition and by Strong Party Identification. Similarly, Strong Party Identification appears unrelated to deciding to drop an alter. And Figure S18 shows how the proportion dropping alters varies by experimental condition and by participant gender. Compared to the control condition, men seem more likely to drop others in the experimental conditions. Table S10 presents six mixed models predicting whether the participant dropped an alter. Model 1 shows that there is not a main effect of participant politics and that it does not interact with the effect of same party, and Model 2 shows that the effect of condition on dropping alters does not vary by whether the participant identifies as a Democrat or Republican. Similarly, Model 3 shows that there is no main effect of Strong Party Identification nor do Strong Party Identifiers moderate the effect of same party, and Model 4 shows that the effect of condition on dropping alters does not vary by Strong Party Identification. That is, political orientation and whether participants are Strong Party Identifiers do not modify our results for whether to drop an alter. Model 5 shows that there is not a main effect of participant gender nor does gender moderate the effect of same party. In Model 6, we find that two of the three terms for the interaction between gender and condition is significant: men are more likely to drop an alter in the parochial condition and in the undifferentiated condition than they are in the control condition.

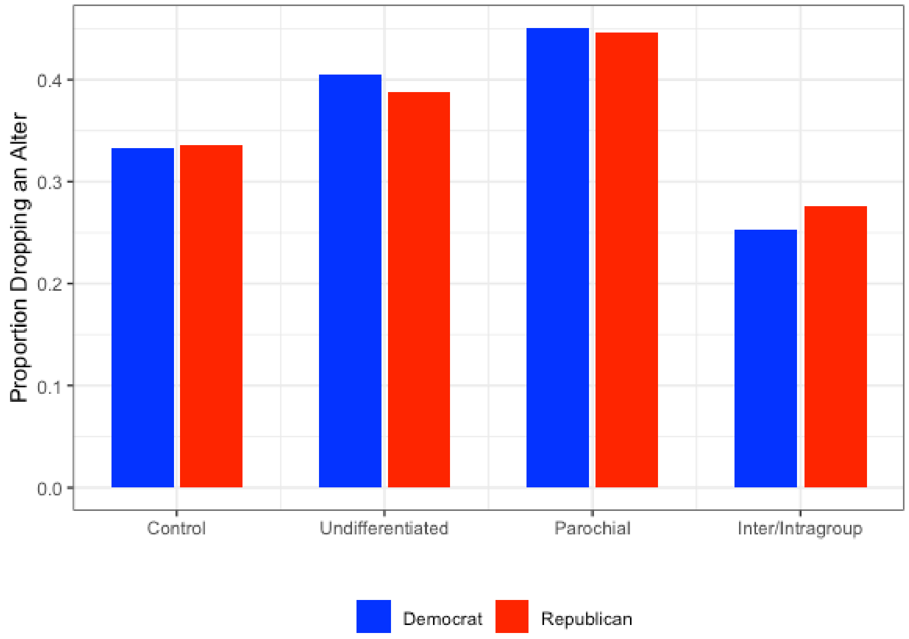

Figure S16: Proportion of dropped alters by experimental condition and participant political orientation.

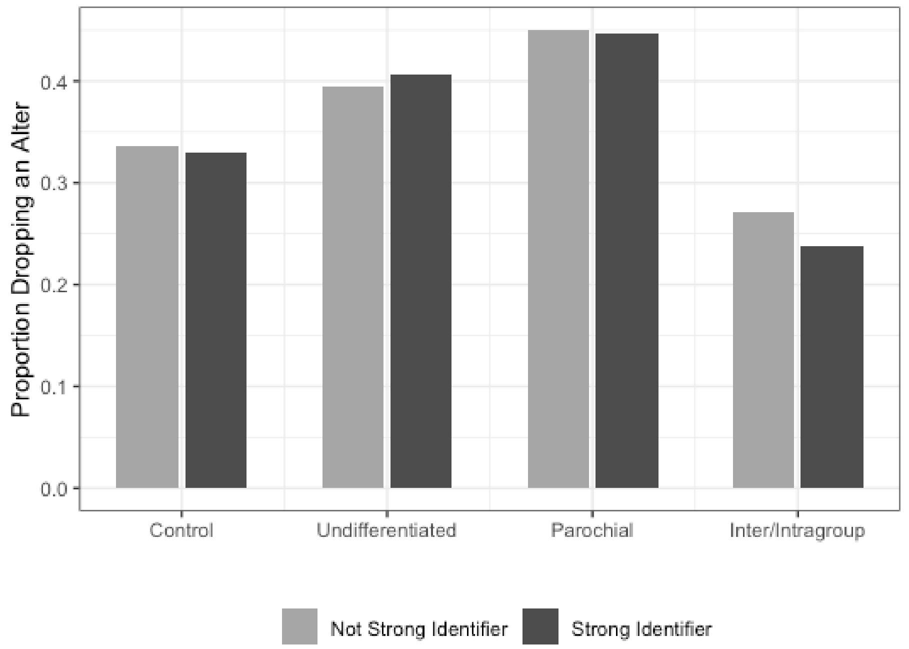

Figure S17: Proportion of dropped alters by experimental condition and Strong Party Identifiers.

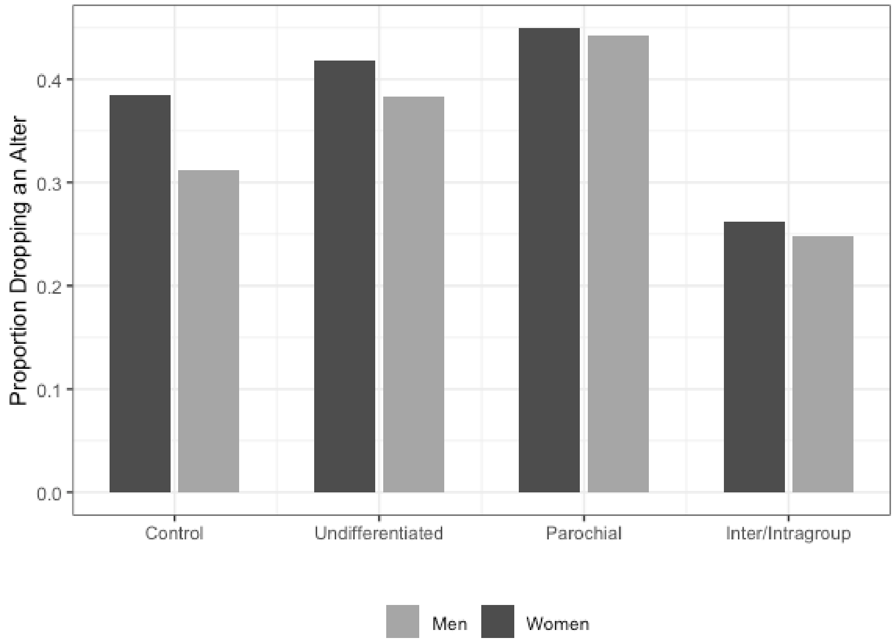

Figure S18: Proportion of dropped alters by experimental condition and participant gender.

Table S10: Mixed effects logistic regression models predicting whether the participant dropped an alter. Permutation-based *p*-value is reported in parentheses.

	Model 1	Model 2	Model 3	Model 4	Model 5	Model 6
Republican Participant (R)	-.290	-.268				
	(.047)	(.083)				
Strong Party Identifier (SPI)			-.122	.081		
			(.352)	(.283)		
Male Participant (M)					.028	-.506
					(.490)	(.004)
Parochial Reputations (P)[1]	.144	.155	.132	.128	.162	-.187
	(.999)	(.999)	(.999)	(.999)	(.999)	(<.001)
Inter/Intragroup Reputations	-.580	-.614	-.591	-.508	-.590	-.705
(I)[1]	(.049)	(.128)	(.029)	(.623)	(.061)	(.150)
Undifferentiated (C)[1]	-.063	-.108	-.078	-.019	-.024	-.212
	(<.001)	(<.001)	(<.001)	(.002)	(<.001)	(<.001)
Same Party (S)	-.411	-.343	-.269	-.194	-.068	-.221
	(.015)	(.012)	(.048)	(.058)	(.278)	(.037)
R, SPI, or M × S	.180		.199		-.276	
	(.238)		(.321)		(.206)	
R, SPI, or M × P		.000		.038		.698
		(.526)		(.494)		(.001)
R, SPI, or M × I		.120		-.212		.272
		(.726)		(.183)		(.189)
R, SPI, or M × C		.154		-.150		.411
		(.758)		(.267)		(.049)
Number of Alters	.373	.373	.373	.373	.380	.384
	(<.001)	(<.001)	(<.001)	(<.001)	(<.001)	(<.001)
Amount Received	-1.340	-1.340	-1.334	-1.333	-1.373	-1.379
	(<.001)	(<.001)	(<.001)	(<.001)	(<.001)	(<.001)
Amount Given	-.250	-.250	-.251	-.251	-.221	-.213
	(.001)	(.001)	(.001)	(.001)	(.005)	(.009)
Following Round 8[2]	-1.131	-1.128	-1.135	-1.135	-1.122	-1.125
	(.999)	(.999)	(.999)	(.999)	(.999)	(.999)
Following Round 12[2]	-1.890	-1.886	-1.896	-1.896	-1.914	-1.916
	(.999)	(.999)	(.999)	(.999)	(.999)	(.999)
Following Round 16[2]	-2.625	-2.620	-2.631	-2.631	-2.633	-2.637
	(.999)	(.999)	(.999)	(.999)	(.999)	(.999)
Constant	-.565	-.597	-.715	-.791	-.802	-.555
	(<.001)	(<.001)	(<.001)	(<.001)	(<.001)	(<.001)

Note: Inference is based on 1,000 permutations of the outcome within network rounds. [1]Reference category is the Control condition. [2]Following Round 4 is the reference category. N = 3,651 decisions nested in participants in networks. All tests are two-tailed.

Which Ties were Severed?

Figure S19 shows descriptive statistics for those who were dropped by participant political identity and experimental condition. In the control condition, we see that Democratic participants are slightly more likely to drop someone with the same political affiliation while Republican participants are slightly less likely to do so. This skew is due to the population being 67% democrats; if the population were 50/50 Democrats and Republicans we would expect equal proportions for the control condition. But in all experimental conditions where politics were shown, we see participants are less likely to drop ingroup members. The sole exception to this pattern is Republicans in the parochial reputations condition. Importantly, however, Model 1 in Table S11 shows that the effect of sharing a political party by experimental condition is not significantly different by participant political affiliation (i.e., none of the terms associated with the three-way interaction are significant).

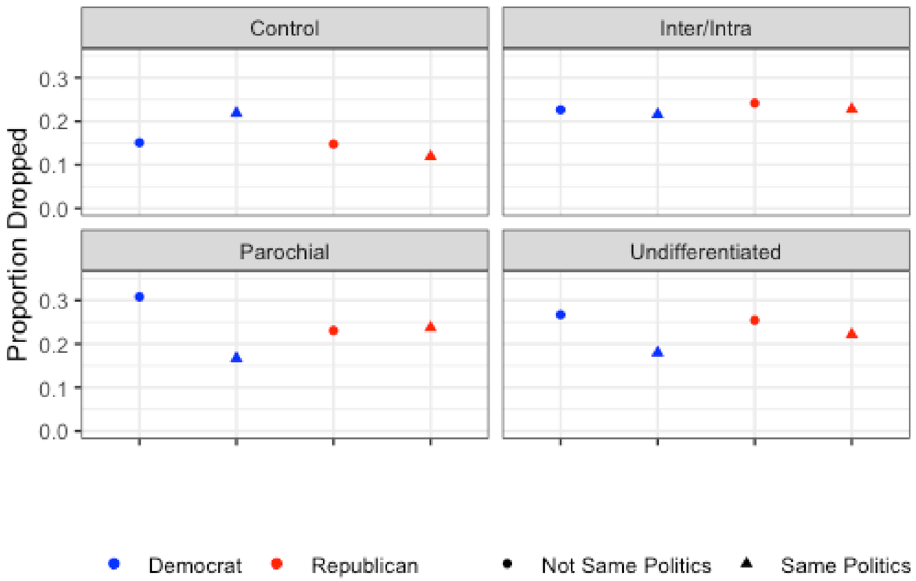

Figure S19: Descriptive statistics for alters who were dropped by participant politics and whether alter shared the same political orientation. Each condition shown.

Figure S20 shows descriptive statistics for those who were dropped by experimental condition and whether the participant is a Strong Party Identifier. In the intra/intergroup condition, Strong Party Identifiers are less likely to drop others who do not share a political affiliation, but otherwise the patterns are stable. Model 2 in Table S11 shows that the interaction between male participants, same politics, and the intra/intergroup condition is negative and significant, meaning that the effect of same politics is significantly weaker among Strong Party Identifiers versus those who are not Strong Party Identifiers in the intra/intergroup condition. We would expect this to be a positive effect if Strong Party Identifiers really rely on politics more than cooperation.

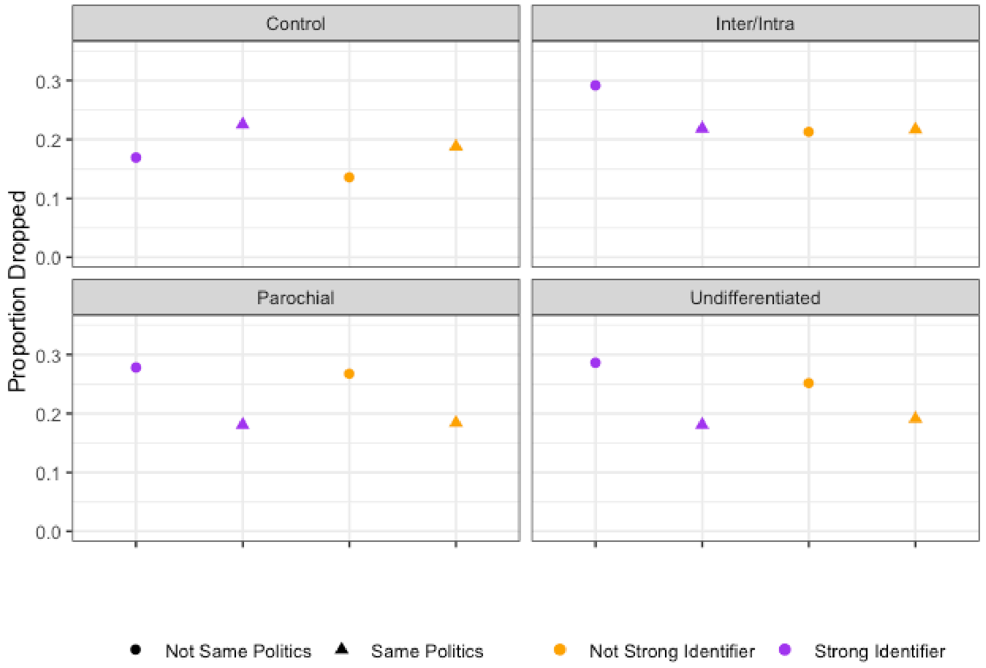

Figure S20: Descriptive statistics for alters who were dropped by whether participants are Strong Party Identifiers and whether alter shared the same political orientation. Each condition shown.

Finally, Figure S21 shows descriptive statistics for those who were dropped by participant gender and experimental condition. Here the descriptive statistics illustrate the same patterns by gender, and the statistical model (Model 3 in Table S11) shows no significant interaction effects.

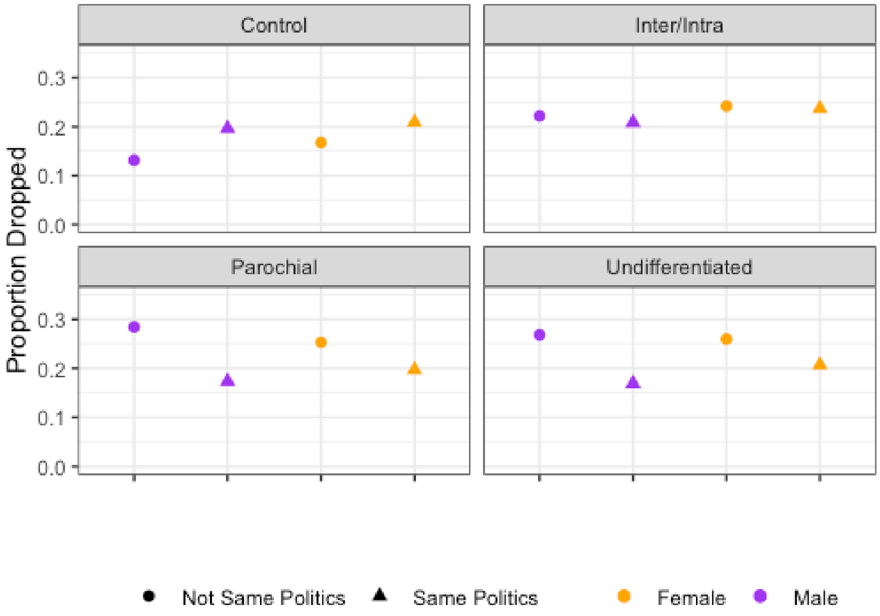

Figure S21: Descriptive statistics for alters who were dropped by participant gender and whether alter shared the same political orientation. Each condition shown.

Table S11: Summary of conditional logistic regression models predicting which alter the participant dropped. Robust standard errors are reported in brackets and parametric *p*-values are reported in parentheses.

	Controlling for:					
	Republican		Strong Party Identifier (SPI)		Male	
	Log Odds [Robust S.E.] Lower 95% C.I.	Odds Ratio (*p*-value) Upper 95% C.I.	Log Odds [Robust S.E.] Lower 95% C.I.	Odds Ratio (*p*-value) Upper 95% C.I.	Log Odd [Robust S.E.] Lower 95% C.I.	Odds Ratio (*p*-value) Upper 95% C.I.
Alter Endowment	.332 [.040]	1.394 (<.001)	.331 [.040]	1.393 (<.001)	.320 [.041]	1.377 (<.001)
	.253 – .410		.252 – .410		.240 – .400	
Received from Alter	-.049 [.003]	.953 (<.001)	-.048 [.003]	.953 (<.001)	-.048 [.003]	.953 (<.001)
	-.055 – -.042		-.054 – -.042		-.054 – -.041	
Same Party (S)	.236 [.304]	1.266 (.296)	-.021 [.289]	.979 (.398)	.121 [.309]	1.129 (.370)
	-.361 – .832		-.588 – .545		-.485 – .727	
Average Amount Given	-.050 [.005]	.951 (<.001)	-.051 [.005]	.950 (<.001)	-.052 [.005]	.950 (<.001)
	-.060 – -.040		-.061 – -.042		-.062 – -.041	
S × Republican, SPI, or Male Participants	-.076 [.418]	.926 (.392)	.499 [.460]	1.647 (.222)	.227 [.455]	1.255 (.352)
	-.896 – .744		-.403 – 1.401		-.664 – 1.119	
S × Parochial Reputations	-1.298 [.370]	.273 (.001)	-.747 [.355]	.475 (.045)	-.944 [.392]	.389 (.022)
	-2.024 – -.572		-1.440 – -.048		-1.712 – -.177	
S × Undifferentiated	-.751 [.368]	.472 (.050)	-.419 [.347]	.658 (.193)	-.461 [.395]	.631 (.202)
	-1.473 – -.029		-1.100 – .261		-1.235 –.312	
S × Inter/Intragroup Reputations	-.824 [.398]	.439 (.047)	-.072 [.369]	.930 (.391)	-.262 [.468]	.769 (.341)
	-1.604 – -.045		-.795 – .650		-1.179 – .654	
S × Parochial Reputations × Republican, SPI, Male Participants	.921 [.526]	2.513 (.086)	-.291 [.563]	.747 (.349)	.050 [.557]	1.051 (.397)
	-.109 – 1.952		-1.394 – .812		-1.042 – 1.141	
S × Undifferentiated × Republican, SPI, Male Participants	.537 [.547]	1.711 (.247)	-.268 [.598]	.765 (.361)	-.400 [.568]	.670 (.311)
	-.536 – 1.610		-1.440 – -.048		-1.514 – .714	
S × Inter/Intragroup Reputations × Republican, SPI, Male Participants	.873 [.585]	2.395 (.131)	-1.613 [.727]	.199 (.034)	-.412 [.640]	.662 (.324)
	-.272 – 2.019		-3.038 – -.189		-1.667 – .843	

Note: All tests are two-tailed.

New Alter Selections

In terms of who participants selected for new ties, Figure S22 shows the proportion of selected others who share the same political orientation as the participant by participants political affiliation and experimental condition. In all three experimental conditions the proportion of ingroup others selected increases, which is evidence of political homophily. As shown in Table S12, after controlling for alter endowments and reputations, we find that Republican participants are more likely to select other Republicans in the parochial condition only. It is worth noting that the main effect of same party is still significant in that model. That is, it is still the case that participants in the parochial reputations condition rely on homophily to form new ties: it is just slightly stronger for Republicans in this one experimental condition.

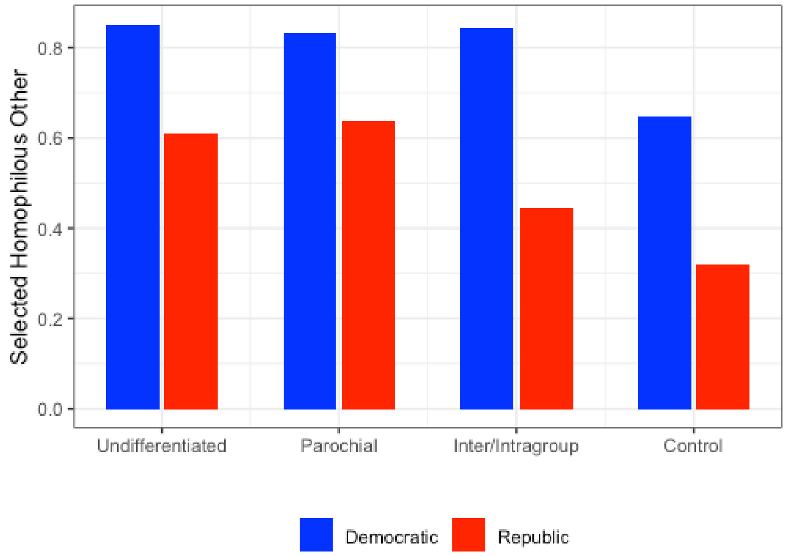

Figure S22: Proportion of times the participant selected a new alter who shared their political affiliation. Color denotes political orientation. Each condition shown.

Table S12: Summary of conditional logistic regression models predicting with whom the participant chose to form a tie. Permutation-based *p*-value is reported in parentheses.

	Control	Undifferentiated	Parochial	Inter/Intragroup Reputations
Alter Endowment	.357<br>(<.001)	.099<br>(.033)	0.240<br>(<.001)	0.275<br>(<.001)
Average Given to Everyone	.083<br>(<.001)	.068<br>(<.001)		
Average Given to Ingroup Members			0.063<br>(<.001)	0.037<br>(.001)
Average Given to Outgroup Members				0.076<br>(<.001)
Homophily (H)	-.161<br>(.162)	1.260<br>(<.001)	1.183<br>(<.001)	0.968<br>(<.001)
H × Republican Participant	.432<br>(.090)	-.095<br>(.333)	.566<br>(.007)	-.022<br>(.499)

Note: Inference is based on 1,000 permutations of the outcome within network-rounds. All tests are two-tailed.

Figure S23 shows the proportion of selected others who share the same political orientation as the participant by experimental condition and whether participants rated themselves as Strong Party Identifiers. The descriptive patterns do not identify any particularly problematic or concerning conditions. Results of a conditional logistic regression (Table S13) show that after controlling for endowment and how much alters gave, we observe no statistically significant homophily effect in the control condition, and positive and statistically significant effects in all experimental conditions, but the positive effect in the undifferentiated condition is stronger for Strong Party Identifiers.

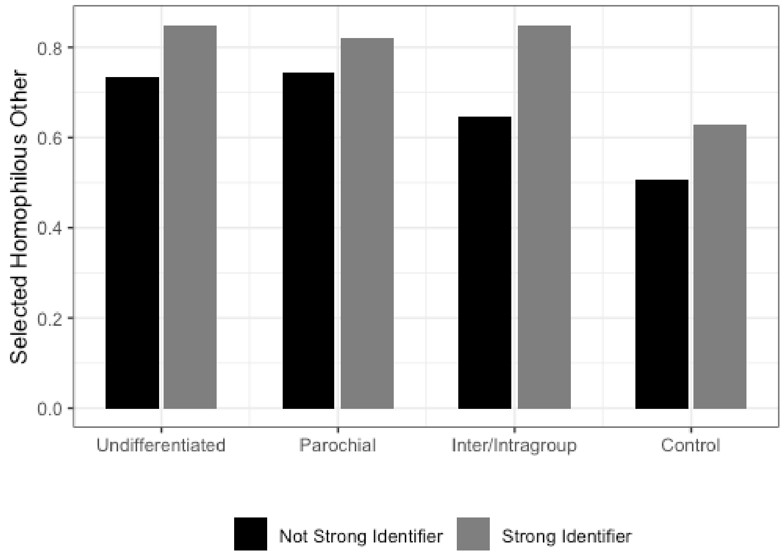

Figure S23: Proportion of times the participant selected a new alter who shared their political affiliation. Color denotes whether the participant is a Strong Party Identifier. Each condition shown.

Table S13: Summary of conditional logistic regression models predicting with whom the participant chose to form a tie. Permutation-based *p*-value is reported in parentheses.

	Control	Undifferentiated	Parochial	Inter/Intragroup Reputations
Alter Endowment	.369<br>(<.001)	.100<br>(.032)	.234<br>(<.001)	.278<br>(<.001)
Average Given to Everyone	.084<br>(<.001)	.068<br>(<.001)		
Average Given to Ingroup Members			.063<br>(<.001)	.037<br>(.001)
Average Given to Outgroup Members				.075<br>(<.001)
Same Party (S)	-.185<br>(.167)	1.098<br>(<.001)	1.357<br>(<.001)	.630<br>(.004)
S × Strong Party Identifier	.293<br>(.180)	.469<br>(.023)	.291<br>(.106)	1.126<br>(.004)

Note: Inference is based on 1,000 permutations of the outcome within network-rounds. All tests are two-tailed.

Figure S24 shows the proportion of selected others who share the same political orientation as the participant by gender and experimental condition. As with Strong Party Identification, the descriptive patterns do not identify any particularly problematic or concerning conditions. Results of a conditional logistic regression (Table S14) show that after controlling for endowment and how much alters gave, the homophily effect is weaker for men in the inter/intra

group condition. Here we observe no homophily effect in the control condition, and positive effects in all experimental conditions, but that positive effect is weaker among men in the intra/intergroup condition.

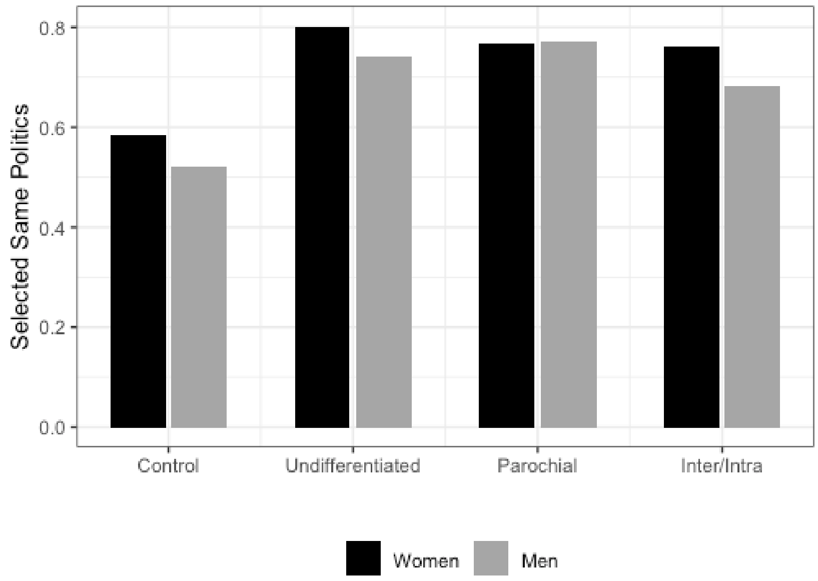

Figure S24: Proportion of times the participant selected a new alter who shared their political affiliation. Color denotes participant gender. Each condition shown.

Table S14: Summary of conditional logistic regression models predicting with whom the participant chose to form a tie. Permutation-based *p*-value is reported in parentheses.

	Control	Undifferentiated	Parochial	Inter/Intragroup Reputations
Alter Endowment	.369<br>(<.001)	.110<br>(.018)	.232<br>(<.001)	.306<br>(<.001)
Average Given to Everyone	.083<br>(<.001)	.068<br>(<.001)		
Average Given to Ingroup Members			.062<br>(<.001)	.034<br>(.001)
Average Given to Outgroup Members				.079<br>(<.001)
Same Party (S)	-.091<br>(.322)	1.193<br>(<.001)	1.286<br>(<.001)	1.578<br>(<.001)
S × Male Participant	.101<br>(.366)	.042<br>(.397)	.283<br>(.110)	-.949<br>(.005)

Note: Inference is based on 1,000 permutations of the outcome within network-rounds. All tests are two-tailed.

Accepting Tie Requests

Table S15 presents three mixed models predicting whether respondents accepted tie requests. Model 1 shows that the homophily effect is not significantly different by participant politics, nor experimental condition. Model 2 includes Strong Party Identification instead of political affiliation and finds the same patterns of no statistically significant moderation. Finally, Model 3 includes participant gender and its interaction with homophily and experimental condition. Again, we observe no statistically significant moderation. Participant politics, Strong Party Identification, and gender do not significantly moderate decisions to drop alters.

Table S15: Summary of mixed effects logistic regression model predicting whether the respondent accepted tie requests. Multiple requests nested in participants, and participants nested in networks. Permutation-based *p*-value is reported in parentheses.

Following Round 8[1]	-.072	-.078	.013
	(.482)	(.457)	(.329)
Following Round 12[1]	-.373	-.361	-.308
	(.068)	(.081)	(.128)
Following Round 16[1]	-.119	-.101	-.066
	(.444)	(.460)	(.509)
Average Given to Alters	.085	.084	.084
	(<.001)	(<.001)	(<.001)
Republican Participant (R)	-.395		
	(.195)		
Strong Party Identification (SPI)		-.175	
		(.457)	
Male Participant (M)			-.056
			(.397)
Same Party (S)	-.222	.051	-.022
	(.237)	(.427)	(.488)
Parochial Reputations[2] (P)	-.751	-1.060	-.031
	(.132)	(.037)	(.695)
Inter/Intragroup Reputations[2] (I)	.101	.104	.995
	(.377)	(.240)	(.068)
No Politics/Control[2] (C)	-.225	-.270	-.282
	(.458)	(.382)	(.326)
S × P	.567	.703	-.195
	(.076)	(.049)	(.413)
S × I	-.403	-.704	-.358
	(.236)	(.117)	(.258)
S × C	-.109	-.233	-.087
	(.415)	(.284)	(.438)
R, SPI, or M × S	.890	.095	.367
	(.071)	(.551)	(.319)
R, SPI, or M × P	-.047	.715	-1.097
	(.566)	(.192)	(.067)
R, SPI, or M × I	-.032	-.199	-.988
	(.545)	(.281)	(.171)
R, SPI, or M × C	.374	.819	.576
	(.373)	(.180)	(.176)
R, SPI, or M × S × P	-.291	-.475	.934
	(.316)	(.341)	(.097)
R, SPI, or M × S × I	.211	1.138	-.367
	(.489)	(.087)	(.390)

R, SPI, or M × S × C	-.307<br>(.366)	-.495<br>(.418)	-.580<br>(.259)
Constant	-1.373<br>(<.001)	-1.469<br>(<.001)	-1.603<br>(<.001)

Note: Inference is based on 1,000 permutations of the outcome within networks. [1]Following Round 4 is the reference category. [2]Undifferentiated is the reference category. All tests are two-tailed.

Becoming isolated from networks

Figures S25-27 show the count of isolates by political affiliation, Strong Party Identification, and participant gender, respectively. Figure S25 shows that Republicans become isolated in the intra/intergroup condition at the same rate as the control, which is less than in the other two experimental conditions. This pattern is statistically significant, as Table S16 shows a significant interaction between Republican participants and the intra/intergroup condition, meaning that Republicans are less likely to be isolated in the inter/intra group condition. The other factors (i.e., Strong Party Identification and gender) are not significantly associated with isolation and do not moderate the effect of condition on isolation.

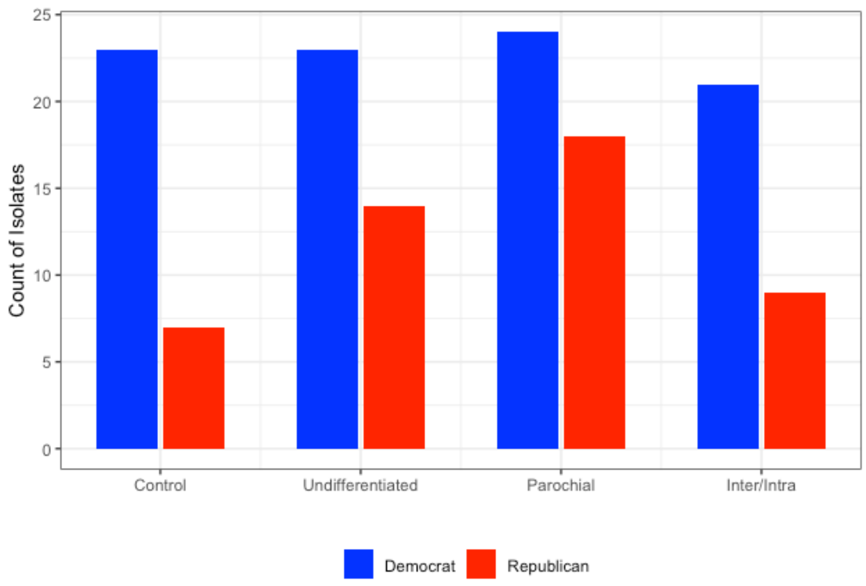

Figure S25: Count of participants who became isolated from the network by experimental conditions. Color denotes participant political orientation.

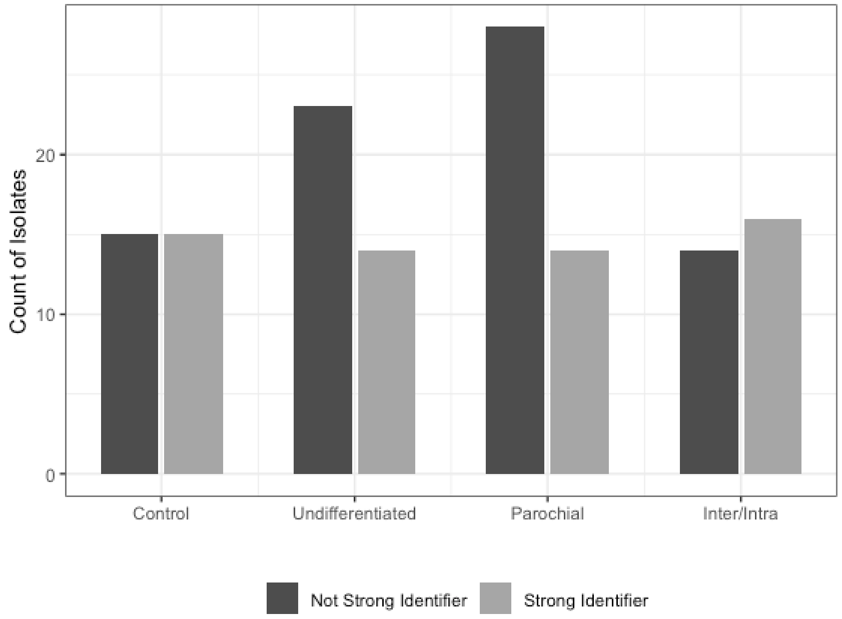

Figure S26: Count of participants who became isolated from the network by experimental conditions. Color denotes whether the participant is a Strong Party Identifier.

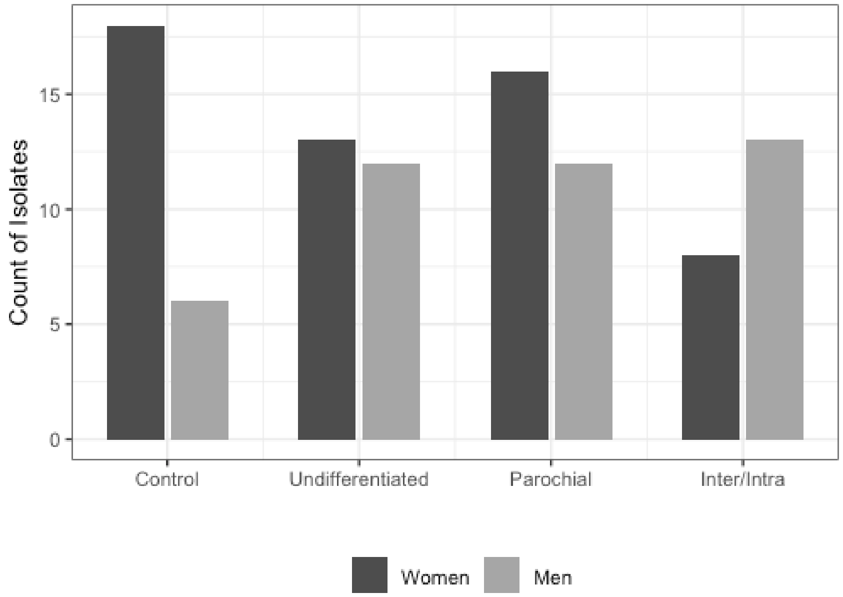

Figure S27: Count of participants who became isolated from the network by experimental conditions. Color denotes participant gender.

Table S16: Summary of Cox Proportional Hazard Model predicting network isolation. Permutation-based *p*-value is reported in parentheses.

Endowment[1]	-.001<br>(<.001)	-.001<br>(<.001)	-.001<br>(<.001)
Average Given[1]	-.087<br>(<.001)	-.087<br>(<.001)	-.099<br>(<.001)
Parochial Reputations[2] (P)	.034<br>(.695)	-.081<br>(.042)	.349<br>(.134)
Inter/Intragroup Reputations[2] (I)	.552<br>(<.001)	.170<br>(.045)	.140<br>(.096)
No Politics/Control[2] (C)	.194<br>(.033)	.138<br>(.114)	.426<br>(.010)
Republican Participant (R)	.025<br>(.468)		
Strong Party Identification (SPI)		.004<br>(.662)	
Male Participant (M)			-.124<br>(.524)
P × R, SPI, or M	.073<br>(.453)	.452<br>(.087)	-.452<br>(.112)
I × R, SPI, or M	-.869<br>(.045)	.171<br>(.328)	.257<br>(.433)
C × R, SPI, or M	.119<br>(.381)	.138<br>(.349)	-.304<br>(.224)

Note: Inference is based on 1,000 permutations of time to isolation within networks. [1]Time-varying covariate. [2]Undifferentiated reputations is the reference category. Robust standard errors reported, clustered on networks. All tests are two-tailed.

Supplementary Note 2

In this section, we visualize all 40 of our networks at the conclusion of the study. In Figures S28-31, node color represents political orientation, node size is proportional to endowments, connections denote opportunities to engage in repeated prisoner's dilemma interactions, and the network-level segregation coefficient is reported at the top right of each network.

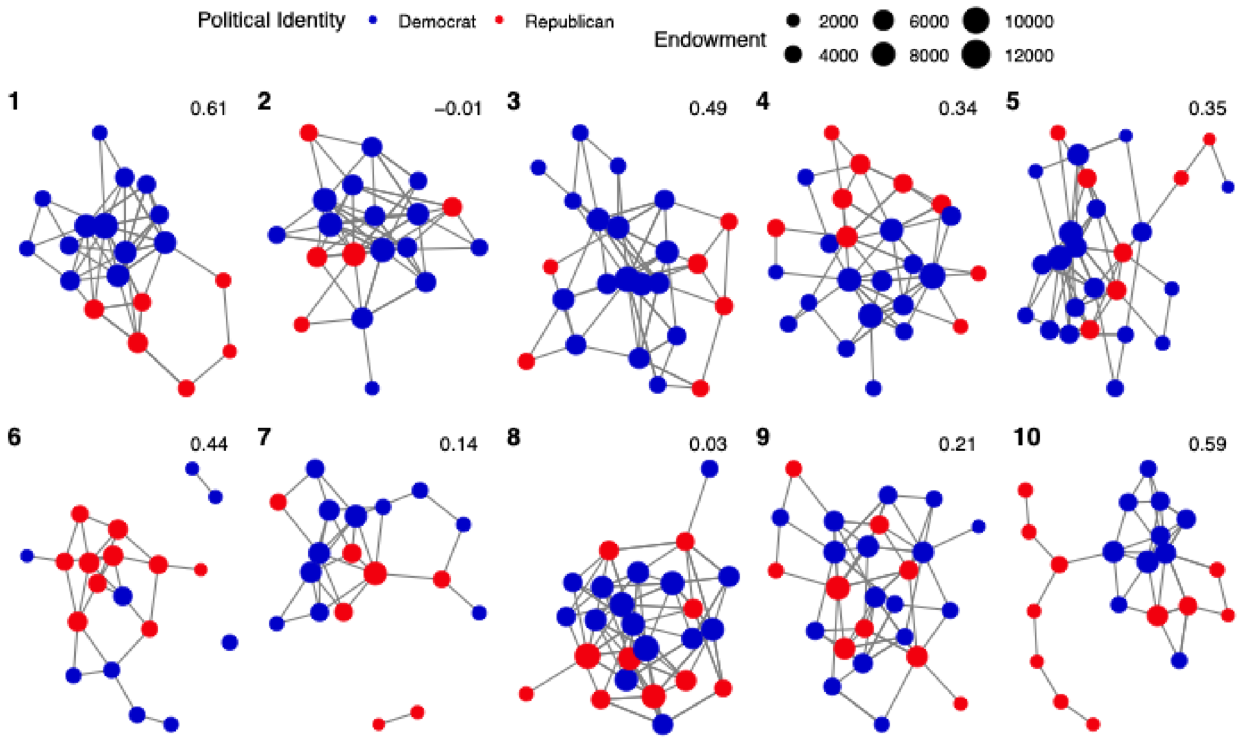

Figure S28: All ten networks in the control condition at the end of the study. Politics were unknown in this condition. Node size is proportional to endowment and nodes are colored according to political identification. Network political segregation reported to the upper-right of each network.

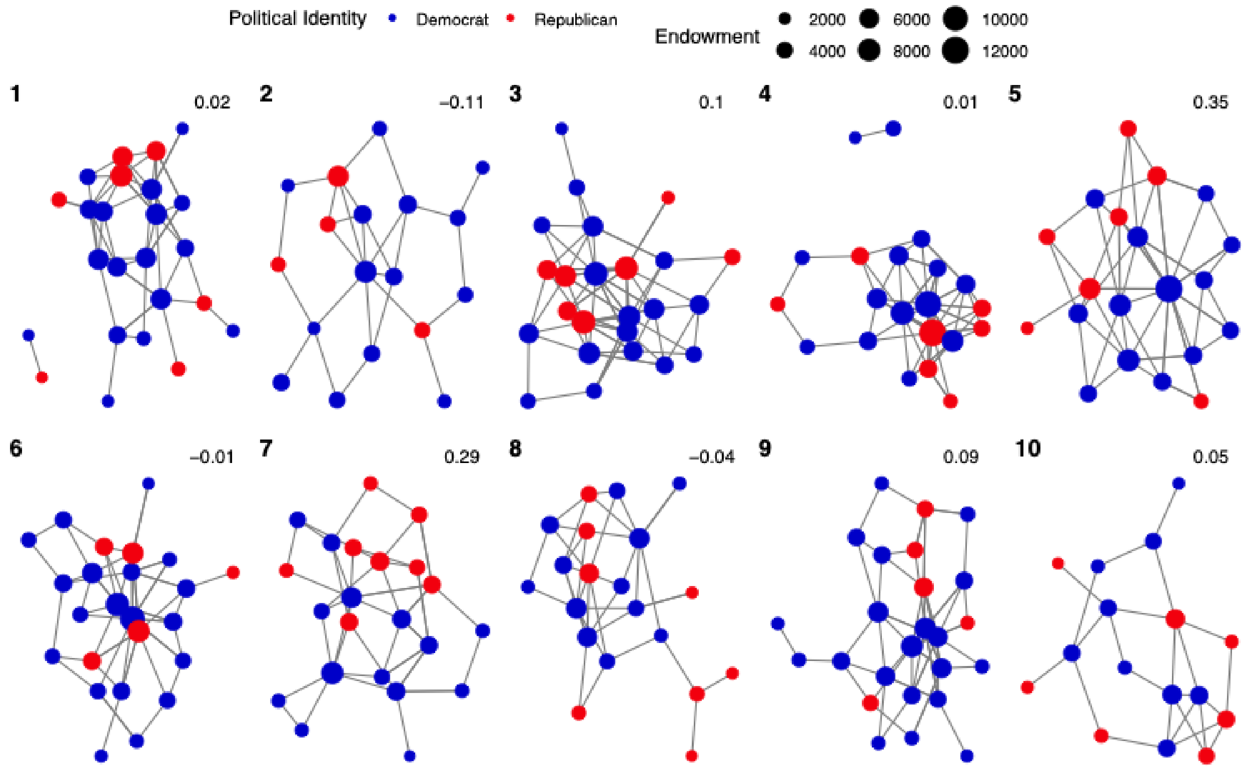

Figure S29: All ten networks in the undifferentiated reputation condition at the end of the study. Politics were known in this condition. Node size is proportional to endowment and nodes are colored according to political identification. Network political segregation reported to the upper-right of each network.

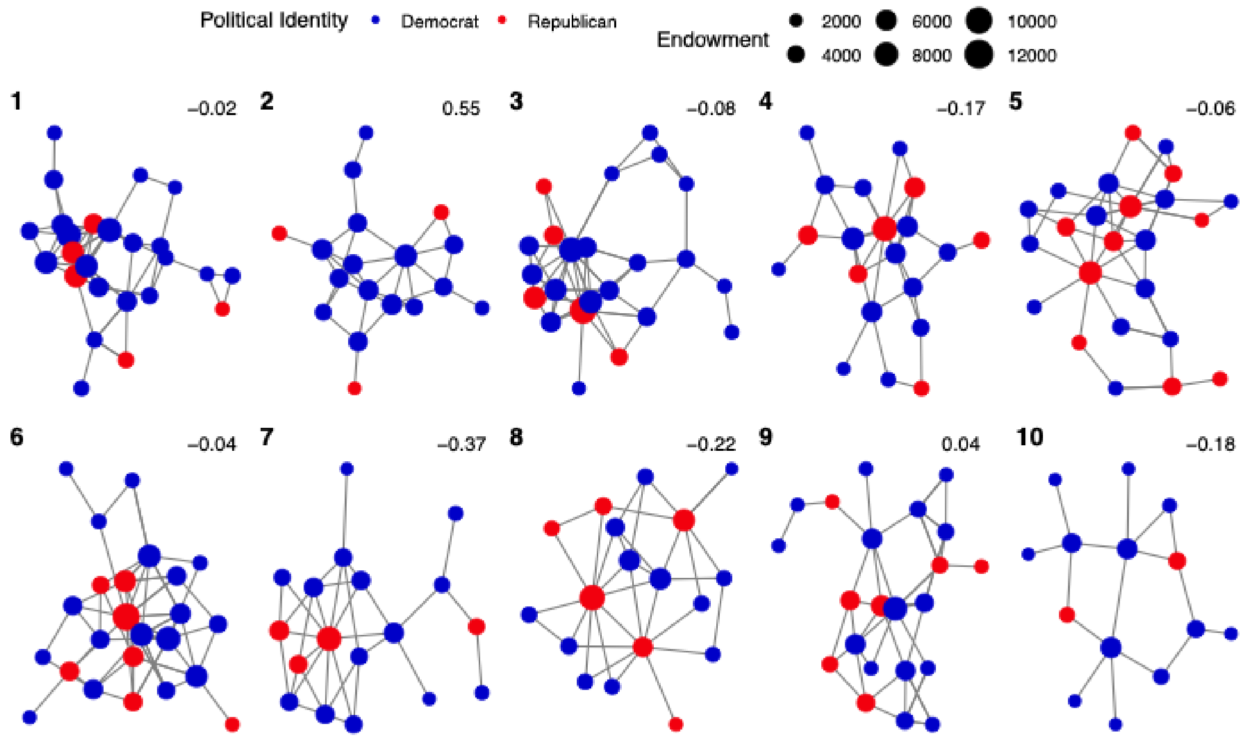

Figure S30: All ten networks in the intra/intergroup reputation condition at the end of the study. Politics were known in this condition. Node size is proportional to endowment and nodes are colored according to political identification. Network political segregation reported to the upper-right of each network.

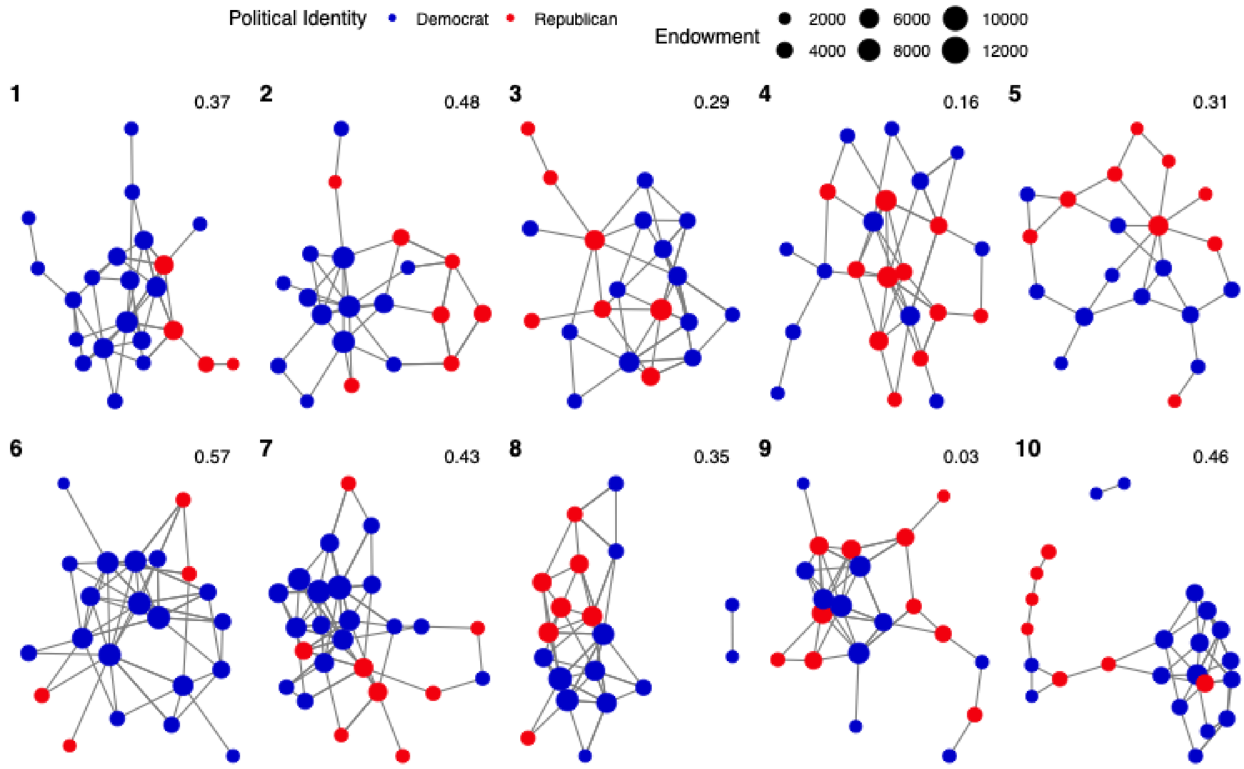

Figure S31: All ten networks in the parochial reputation condition at the end of the study. Politics were known in this condition. Node size is proportional to endowment and nodes are colored according to political identification. Network political segregation reported to the upper-right of each network.
