# Supplementary material for: Reputations for treatment of outgroup members can prevent the emergence of political segregation in cooperative networks
Source: Nat Commun. 2023 Nov 24;14:7721. doi: 10.1038/s41467-023-43486-7 (PMC10674010; doi:10.1038/s41467-023-43486-7)
Supplement: Supplementary file 3 — Reporting Summary [file 41467_2023_43486_MOESM3_ESM.pdf]

nature portfolio

Corresponding author(s): Brent Simpson and David Melamed

Last updated by author(s): Oct 4, 2023

# Reporting Summary

Nature Portfolio wishes to improve the reproducibility of the work that we publish. This form provides structure for consistency and transparency in reporting. For further information on Nature Portfolio policies, see our Editorial Policies and the Editorial Policy Checklist.

## Statistics

For all statistical analyses, confirm that the following items are present in the figure legend, table legend, main text, or Methods section.

| n/a | Confirmed | |
|---|---|---|
| ☐ | ☒ | The exact sample size (*n*) for each experimental group/condition, given as a discrete number and unit of measurement |
| ☐ | ☒ | A statement on whether measurements were taken from distinct samples or whether the same sample was measured repeatedly |
| ☐ | ☒ | The statistical test(s) used AND whether they are one- or two-sided<br>*Only common tests should be described solely by name; describe more complex techniques in the Methods section.* |
| ☐ | ☒ | A description of all covariates tested |
| ☐ | ☒ | A description of any assumptions or corrections, such as tests of normality and adjustment for multiple comparisons |
| ☐ | ☒ | A full description of the statistical parameters including central tendency (e.g. means) or other basic estimates (e.g. regression coefficient) AND variation (e.g. standard deviation) or associated estimates of uncertainty (e.g. confidence intervals) |
| ☐ | ☒ | For null hypothesis testing, the test statistic (e.g. $F$, $t$, $r$) with confidence intervals, effect sizes, degrees of freedom and $P$ value noted<br>*Give P values as exact values whenever suitable.* |
| ☒ | ☐ | For Bayesian analysis, information on the choice of priors and Markov chain Monte Carlo settings |
| ☐ | ☒ | For hierarchical and complex designs, identification of the appropriate level for tests and full reporting of outcomes |
| ☐ | ☒ | Estimates of effect sizes (e.g. Cohen's $d$, Pearson's $r$), indicating how they were calculated |

*Our web collection on statistics for biologists contains articles on many of the points above.*

## Software and code

Policy information about availability of computer code

| | |
|---|---|
| Data collection | We used the Breadboard software (version 2.3.1), which is available freely online at the Yale networks center. |
| Data analysis | We used R for data analyses. R scripts to replicate our results are deposited with OSF (https://osf.io/xkamd/), along with the original data. |

For manuscripts utilizing custom algorithms or software that are central to the research but not yet described in published literature, software must be made available to editors and reviewers. We strongly encourage code deposition in a community repository (e.g. GitHub). See the Nature Portfolio guidelines for submitting code & software for further information.

## Data

Policy information about availability of data

All manuscripts must include a data availability statement. This statement should provide the following information, where applicable:

- Accession codes, unique identifiers, or web links for publicly available datasets
- A description of any restrictions on data availability
- For clinical datasets or third party data, please ensure that the statement adheres to our policy

The processed data, along with distributions of regression coefficients that were used for non-parametric inference, have been deposited with OSF: https://osf.io/xkamd/ All results, tables and figures presented in the Main text and Supplementary Information file can be reproduced with these data. The raw data are available upon request from the third author.

## Research involving human participants, their data, or biological material

Policy information about studies with human participants or human data. See also policy information about sex, gender (identity/presentation), and sexual orientation and race, ethnicity and racism.

| | |
|---|---|
| Reporting on sex and gender | We did not make any gender-based predictions, but we did measure participant gender. As such, we include sensitivity analyses in our SI on how results vary by gender. |
| Reporting on race, ethnicity, or other socially relevant groupings | We did not make any predictions about race ethnicity or other groupings. The study focuses on political segregation, so we do report on democrats versus republicans in the US context, both in the main text and in the SI. |
| Population characteristics | The sample was 37.9 years old, on average, with a standard deviation of 12.57 years. The sample was also 50.2% female. In terms of race/ethnicity the sample was 79% white, 8% Asian, 5% black, 6% Latino and 2% other/mixed race. |
| Recruitment | Participants from Prolific that were randomly assigned to experimental conditions. Qualtrics maintains the sample. Systematic comparisons of crowdsource samples to representative ones tend to find that they are younger and more technologically literature than the general population.<br>Importantly, however, these participants were randomly assigned to experimental conditions. |
| Ethics oversight | The University of South Carolina |

Note that full information on the approval of the study protocol must also be provided in the manuscript.

# Field-specific reporting

Please select the one below that is the best fit for your research. If you are not sure, read the appropriate sections before making your selection.

☐ Life sciences ☒ Behavioural & social sciences ☐ Ecological, evolutionary & environmental sciences

For a reference copy of the document with all sections, see nature.com/documents/nr-reporting-summary-flat.pdf

# Behavioural & social sciences study design

All studies must disclose on these points even when the disclosure is negative.

| | |
|---|---|
| Study description | We conducted an experiment. Humans were embedded in networks of 20-28 people. We varied characteristics of the networks. The data are quantitative in nature. Our web application tracked the behaviors of participants. We then wrangled those data into something amenable for statistical/quantitative analyses. |
| Research sample | Prolific workers from the United States. They are not representative, but Prolific provides a large enough pool to run experiments with networks of participants. The recorded demographics were noted above (age, gender, race). We used Prolific as it was one of only a few crowdsourcing sites with a large enough pool to run the experiment. At the same time, Prolific has the best workers. An alternative is Turk, but the data quality from Turk workers is often questionable. |
| Sampling strategy | We oversampled on Conservatives/Republicans to try to get better representation. Prolific samples participants exhaustively. They sample online, via phone, and via snail mail to get participants into their pool. Participants in our study were those available when we started running a session.<br>In terms of the sample size, we used 10 networks per condition, which is customary in this tradition (i.e., we have used 10 per condition on 3 prior occasions and several studies before ours were based on the same sample size). |
| Data collection | We used Breadboard, a novel app for collecting networked responses. Participants followed a link from Prolific to Breadboard, where they completed the study. At the end of the study, we gave them a code to paste in Prolific for payment. The Research Assistant would turn on the Breadboard app and post a link to Prolific enabling participants to enter the app. The app would wait for 15 minutes to allow participants to populate the study and for them to read instructions. If we didnt have sufficient participants, the sessions was canceled. The research assistant was not blind to experimental conditions, but the researcher could not interact with the participants or alter the study in any way. Once the study was programmed into Breadboard, the only differences between conditions were those embedded in the program. |
| Timing | Data were collected during Spring 2022 and Summer 2022. |
| Data exclusions | No data were excluded. |
| Non-participation | In the missing data section of the SI we describe this in detail. In particular, we note in the Missing Data section that 79 participants dropped out of the study. We do not know why they dropped out. In some cases it may have just been internet connectivity but we cannot know for sure. The breadboard app gives participants 10 seconds to respond and drops them if they do not respond (to ensure that the game can proceed). |
| Randomization | Yes, we used random assignment to conditions. |

# Reporting for specific materials, systems and methods

We require information from authors about some types of materials, experimental systems and methods used in many studies. Here, indicate whether each material, system or method listed is relevant to your study. If you are not sure if a list item applies to your research, read the appropriate section before selecting a response.

## Materials & experimental systems

| n/a | Involved in the study |
|---|---|
| ☒ | ☐ Antibodies |
| ☒ | ☐ Eukaryotic cell lines |
| ☒ | ☐ Palaeontology and archaeology |
| ☒ | ☐ Animals and other organisms |
| ☒ | ☐ Clinical data |
| ☒ | ☐ Dual use research of concern |
| ☒ | ☐ Plants |

## Methods

| n/a | Involved in the study |
|---|---|
| ☒ | ☐ ChIP-seq |
| ☒ | ☐ Flow cytometry |
| ☒ | ☐ MRI-based neuroimaging |
